# Understanding Obesity as a Multisystem Disease: Advancing Research, Redefining Diagnostic Criteria, and Establishing Modern Therapeutic Approaches

**DOI:** 10.3390/nu18142317

**Published:** 2026-07-15

**Authors:** Hala Abdallah, Mohamad Khalil, Laura Mahdi, Gianni Pietragalla, Maria Felicia Faienza, Gabriella Garruti, Piero Portincasa

**Affiliations:** 1 Internal Medicine Clinica Medica “A. Murri”, Department of Precision and Regenerative Medicine and Ionian Area (DiMePRe-J), University of Bari ‘Aldo Moro’, 70121 Bari, Italy; halaabdallah18@gmail.com (H.A.); laura.mahdi@uniba.it (L.M.); g.pietragalla@phd.uniba.it (G.P.); 2 Pediatric Unit, Department of Precision and Regenerative Medicine and Ionian Area (DiMePRe-J), University of Bari ‘Aldo Moro’, 70121 Bari, Italy; mariafelicia.faienza@uniba.it; 3 Section of Internal Medicine, Endocrinology, Andrology and Metabolic Diseases, Department of Precision and Regenerative Medicine and Ionian Area (DiMePRe-J), University of Bari ‘Aldo Moro’, 70121 Bari, Italy; gabriella.garruti@uniba.it

**Keywords:** obesity, semaglutide, diet, anti-obesity medication, GIP/GLP-1/glucagon receptor agonists, metabolic surgery

## Abstract

Obesity is a complex, chronic, relapsing disease that affects nearly all physiological functions and homeostatic mechanisms, extending far beyond mere excess adiposity. Traditional clinical practice often relies on uniform interventions that fail to account for diverse patient phenotypes. This review synthesizes current evidence on personalized obesity management, dietary interventions, and advanced pharmacotherapies aligned with the new 2025 Lancet Commission framework. A comprehensive narrative review was conducted across major biomedical databases to evaluate the efficacy, safety, and mechanisms of metabolic interventions, focusing on phenotype-specific dietary matching, bioactive compounds, and modern multi-receptor glucagon-like peptide-1 (GLP-1)-based therapies across preclinical and clinical intervention tiers in adult and pediatric populations. While specific nutraceutical extracts display promising secondary metabolic benefits, their overall weight-loss efficacy remains modest due to variable clinical data and limited sample sizes. Conversely, high-potency anti-obesity medications demonstrate unprecedented weight-reduction efficacy, with semaglutide 2.4 mg and tirzepatide yielding mean body weight losses of ~14.9% and ~20.9%, respectively. Furthermore, landmark outcome data from the SELECT trial confirms that semaglutide 2.4 mg achieves a significant 20% relative risk reduction in major adverse cardiovascular events (MACE), independent of baseline glycemic status or heart failure. However, clinical extension data reveal rapid weight rebound upon drug cessation, highlighting that obesity requires continuous, long-term therapeutic strategies. Effective obesity care demands an operational shift from traditional, one-size-fits-all treatments to personalized, multi-tiered strategies. Combining phenotype-specific lifestyle modifications with potent, long-term multi-hormonal pharmacotherapies or metabolic surgery optimizes long-term therapeutic durability, systemic metabolic health, and sustained cardioprotection.

## 1. Introduction

The World Health Organization (WHO) has defined obesity as “excess or abnormal fat accumulation that presents a risk to health”, and this condition affects about 800 million people globally [1]. About 40.3% of adults in the USA live with obesity, according to the official August 2021–August 2023 NHAHES data published by the Centers for Disease Control and Prevention (CDC), with obesity-related costs at about $173 billion per year [2]. The global prevalence of both overweight and obesity is continuously increasing in adults, adolescents, and children, has reached epidemic levels, and is currently one of the most pressing public health challenges of the 21st century [3]. This chronic disease involves more than 200 countries around the world [4], and the associated morbidity and mortality are a matter of concern. According to the 2021 Global Burden of Disease (GBD) estimates, overweight and obesity were responsible for approximately 3.71 million deaths and 129 million disability-adjusted life years (DALYs) globally [5]. Over the past two decades, the age-standardized DALY rates attributable to excess body weight have increased by more than 15% and have positioned obesity among the fastest-growing risk factors for non-communicable diseases (NCDs) worldwide [5]. With the increasing prevalence of obesity, the prevalence of the comorbidities associated with obesity has also increased [6]. The rise in obesity directly correlates with the global surge in metabolic and cardiovascular conditions, including type 2 diabetes mellitus (T2DM), hypertension, dyslipidemia, atherosclerotic cardiovascular disease (ASCVD), such as heart disease and stroke, sleep apnea, musculoskeletal disorders, psychological comorbidities, and cancer [7,8]. Additional abnormalities associated with obesity include metabolic dysfunction-associated steatotic liver disease (MASLD) [9,10,11] and cholesterol cholelithiasis in both adults and young populations [12,13,14]. Projections estimate that 1.9 billion adults will live with obesity by 2035 [15,16], and that by 2050, over 1.3 billion individuals globally will be living with diabetes, a burden primarily driven by the ongoing obesity crisis [3]. Concurrently, epidemiological models forecast a doubling in the incidence of cardiovascular events in several countries within the next decade and a surge in obesity-related cancers to over 2 million new cases annually by 2070, representing approximately 7% of the global cancer burden [17]. Beyond the clinical consequences, obesity imposes a profound economic burden. In 2019, global costs, including healthcare expenditures and productivity losses, ranged from $3.19 billion in low-income countries to a staggering $1.33 trillion in high-income nations [17].

This review aims to provide an in-depth exploration of obesity, beginning with a refined definition aligned with the latest international consensus. It examines the multifactorial etiology of obesity, including dietary, genetic, and environmental determinants, and outlines its systemic metabolic consequences. Screening for obesity must include high-risk patients who may not otherwise receive appropriate counselling about health risks, lifestyle changes, therapeutic options, and risk factor reduction. Furthermore, the review critically appraises current strategies for weight management, ranging from dietary interventions and nutritional regimens to pharmacological therapies and bariatric surgery in both pediatric and adult populations.

## 2. Search Strategy and Literature Identification

This review was designed as a comprehensive narrative rather than a formal systematic review. The objective was to capture the breadth of evidence on obesity, including foundational historical work and the most recent advances in pathophysiology, prevention, and management.

A structured literature search was conducted across major biomedical databases, including MEDLINE Complete and Embase, selected to ensure broad coverage of clinical, epidemiological, and translational research. The search strategy was developed collaboratively by all authors and combined both free-text keywords and indexed subject headings (e.g., Medical Subject Headings (MeSH) and Entree terms (Elsevier’s controlled vocabulary). Core search concepts included “obesity,” “overweight,” and “metabolic syndrome”, along with related terms addressing etiology, comorbidities, and therapeutic interventions. The search was primarily focused on titles and abstracts to efficiently identify relevant publications; we found a total of 1254 publications. Exclusions were made for duplicate records, conference abstracts, grey literature, and studies that did not directly investigate the physiological mechanisms or clinical management of adiposity.

While no strict time limits were applied to capture seminal and historically important studies that shaped current understanding of obesity, particular emphasis was placed on recent literature, especially studies reflecting contemporary classifications, guidelines, and therapeutic innovations. Dates from 2020 to 2026 were heavily prioritized to capture the rapid integration of multi-hormonal therapies, the latest 2025 international commission frameworks, and modern diagnostic updates.

This approach allowed the review to contextualize modern findings within the evolution of obesity research. Only peer-reviewed, full-text articles published in English were considered. Priority was given to high-quality evidence, including consensus statements, clinical trials, meta-analyses, and large observational studies relevant to both adult and pediatric populations. Additional articles of significance were identified through manual screening of reference lists from key publications.

As a narrative review, this work is subject to selection bias, as a formal systematic study screening flow diagram or risk-of-bias assessment was not performed. Furthermore, our literature search was restricted to MEDLINE and Embase; the Cochrane Library was not formally searched for intervention updates, which may limit the exhaustiveness of the clinical trial synthesis.

## 3. Obesity

According to the CDC, “weight that is considered higher than what is considered healthy for a given height is described as overweight or obesity” [18]. To first identify obesity, the body mass index (BMI) is used by dividing the weight (kg) by the height (m^2^) [19]. For adults, a BMI of 25.0 to 29.9 kg/m^2^ was defined as overweight, and a BMI of 30 kg/m^2^ or higher was defined as obese [20,21]. The European Society for the Study of Obesity (EASO) recently proposed to consider a new framework for the diagnosis, staging, and management of Obesity in adults. According to this new framework, the diagnosis of visceral obesity must include subjects with a BMI > 25 kg/m^2^ and a waist-to-height ratio > 0.5 associated with medical, functional or psychological comorbidities [11,22].

The evidence of obesity is a call for approaches targeting the prevention and treatment of related comorbidities. Excessive body fat accumulation results from several drivers, which include sustained positive energy balance, i.e., caloric surplus, where caloric intake consistently exceeds the metabolic and physiological energy demands. In addition, interconnected etiological factors include obesogenic environments enriched with hyperpalatable, ultra-processed, hypercaloric foods, sedentary behaviour, gut dysbiosis, genetic background, and epigenetic modifications. Collectively, such drivers disrupt metabolic homeostasis [8,23].

**Figure 1:**
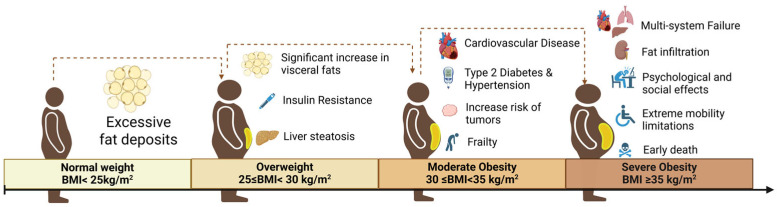
Progression of obesity and its associated metabolic and systemic complications across increasing body mass index (BMI) categories. The continuum begins with normal weight, progressing through overweight, moderate obesity, and severe obesity. Excessive adiposity, particularly visceral fat accumulation, occurs with increasing BMI and is associated with insulin resistance, hepatic steatosis, and several metabolic dysfunctions. Moderate obesity is linked to heightened risk of type 2 diabetes, hypertension, cardiovascular disease, tumor development, and physical frailty [28]. In severe obesity, multisystem complications emerge, including fat infiltration of vital organs, extreme mobility limitations, significant psychological and social burdens, and increased risk of early mortality. This framework highlights the escalating burden of obesity-related pathophysiology with rising adiposity [11].

The body’s energy equilibrium is sustained through coordination of dietary intake, metabolic utilization, and accumulation of energy stores, shaping long-term energy status [24]. Adipose tissue is mainly classified into subcutaneous, visceral (intraperitoneal), and peripheral (subperitoneal) types [25,26]. In case of energy imbalance, the body takes in more energy than it uses, and the excess is stored as energy reserves in new fat cells through adipogenesis [27] (Figure 1).

The optimal metabolic state is governed by the integrated physiological functions of white, brown, and beige adipocytes, each differentially contributing to distinct energy storage and expenditure mechanisms. White adipocytes are distributed throughout the body and primarily serve as the body’s energy reservoir. They facilitate the conversion of circulating glucose and free fatty acids (FFAs) into unilocular lipid droplets composed of triglycerides. These droplets act as storage units that release energy through the controlled efflux of FFAs when metabolic demand increases [29]. Morphologically, white adipocytes are characterized by a single large lipid droplet, relatively low mitochondrial content, and minimal expression of uncoupling protein 1 (UCP1), reflecting their specialized role in energy storage rather than thermogenesis [30,31,32,33]. In contrast, brown adipocytes are enriched with multilocular lipid droplets and a high density of mitochondria, expressing significant levels of UCP1. This protein uncouples oxidative phosphorylation, allowing the dissipation of the proton gradient as heat rather than ATP, which underpins non-shivering thermogenesis. Brown adipocytes are therefore critical for maintaining body temperature and promoting energy expenditure [31]. Beige adipocytes exhibit an intermediate phenotype, capable of adopting brown-like thermogenic activity under specific stimuli such as cold exposure or β-adrenergic activation. Morphologically and functionally, beige adipocytes reside within white adipocyte depots but express UCP1 and possess moderate mitochondrial density when activated, contributing flexibly to both energy storage and thermoregulation [33,34,35,36,37].

Collectively, the metabolic efficiency and adaptability of the body are dependent on the coordinated interactions among white adipocytes, brown adipocytes, and beige adipocytes, which together regulate energy storage, mobilization, and dissipation in response to physiological demands [38,39,40,41]. In addition to its role as a fat storage, adipose tissue is also recognized as a dynamic endocrine organ that secretes adipokines, signalling molecules that play a role in insulin resistance, lipid metabolism, and inflammation. Once dysregulated, these hormones may lead to obesity, cardiometabolic diseases, hypertension, diabetes, and atherosclerosis [42]. For that, a balance between pro-inflammatory and anti-inflammatory adipokines determines whether adipose tissue promotes or protects against cardiometabolic disease [43].

As shown in Table 1, obesity is a heterogeneous condition encompassing multiple phenotypes with distinct clinical and metabolic characteristics.

### 3.1. Metabolically Healthy Obesity (MHO)

The metabolically healthy or benign obese represents 6 to 40% of the total obese population [44]. These patients are characterised by obesity with a lack of cardiometabolic abnormalities [52], less visceral adipose tissue, smaller adipocytes, and a lower inflammatory profile in comparison to the metabolically unhealthy obese subjects [47]. Regardless, a meta-analysis study revealed higher rates of cardiovascular disease, type 2 diabetes, certain cancers, chronic kidney disease, and depressive disorders in children and adults, due to subtle micro-level metabolic abnormalities, including elevated visceral fat, reduced adiponectin, oxidative stress, and chronic inflammation, which are not captured by standard MHO screening criteria [53]. The concept of MHO has also evolved substantially. Although individuals with MHO lack overt metabolic abnormalities at a given time, accumulating longitudinal evidence suggests that this phenotype is frequently transient rather than stable. Recent analyses indicate that most individuals with MHO eventually develop metabolic dysfunction and obesity-related complications during long-term follow-up, supporting the view that MHO represents an earlier stage of disease rather than a benign condition [54]. This concept closely parallels the preclinical obesity category proposed by the 2025 Lancet Commission [55], in which excess adiposity exists without established organ dysfunction but confers increased risk of progression, emphasizing the importance of continued surveillance and early intervention rather than therapeutic inertia.

### 3.2. Metabolically Abnormal Obesity (MAO)

A high number of subjects in the MAO group are overweight or obese with metabolic diseases, i.e., type 2 diabetes, cardiovascular diseases, systolic or diastolic high blood pressure, and a high waist-to-hip ratio. A recent study has shown an increase in oxidative stress, intra-abdominal adiposity, hepatic de novo lipogenesis, cholesterol synthesis, and adipose tissue inflammation, in addition to high levels of non-esterified fatty acids (NEFA), triglycerides, and 24 h plasma glucose in metabolically abnormal obesity [54]. Significant differences exist between MAO and MHO groups in levels of postprandial blood glucose, high-density lipoprotein cholesterol, insulin, and triglycerides [49]. However, common factors also exist between these two groups, such as the increase in arterial stiffness, 24 h plasma glucagon, and subcutaneous abdominal adipose tissue triglyceride synthesis [54].

### 3.3. Sarcopenic Obesity

Sarcopenia is a syndrome characterized by loss of muscle mass, strength, and performance [56]. Individuals living with sarcopenic obesity show an accumulation of body fat and a reduction in lean mass [57], associated with predictive factors such as increased age, decreased physical activity, smoking, low socio-economic status, pulmonary disease, and atherosclerosis [50]. Jiang et al. found an increased risk of CVD, heart disease, and stroke in the sarcopenic obese group compared to the non-sarcopenic group, and the risk becomes higher with a BMI ≥ 28.0 kg/m^2^ [58]. A meta-analysis encompassing 151 studies conducted across countries on every continent, involving a total of 692,056 individuals with an average age of 68.5 years, revealed that the prevalence of sarcopenia varied between 10% and 27% [59]. Another meta-analysis has estimated a prevalence of 9% sarcopenic obesity in both males and females, and its association with metabolic syndromes, CVD, CVD-related mortality, cognitive impairment, lung diseases, and functional limitation [60].

## 4. Factors Contributing to Obesity

**Figure 2:**
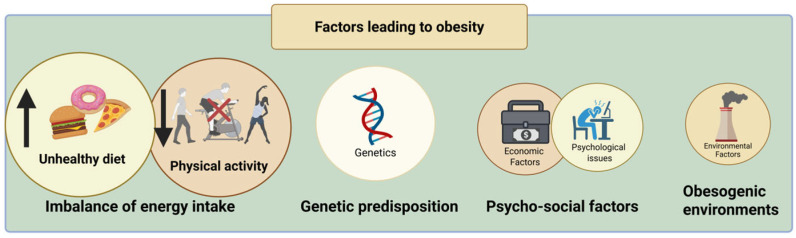
Multifactorial contributors to the development of obesity. An imbalance between energy intake and expenditure is driven by increased consumption of calorie-dense, nutrient-poor foods (unhealthy diet) and reduced levels of physical activity. Genetic predisposition, including inherited traits influencing metabolism and fat storage, plays a critical role [61]. Psycho-social influences, such as socioeconomic status and psychological stressors, contribute to altered eating behaviors and reduced access to healthy lifestyle resources [62]. Additionally, obesogenic environments characterized by limited access to nutritious foods, sedentary lifestyles, and exposure to environmental pollutants further exacerbate obesity risk [63,64].

Obesity is a chronic multifactorial condition that can be influenced by unbalanced dietary habits, lack of physical activity, genetic predisposition, psycho-social, and environmental factors (Figure 2).

The evidence supports the thesis that the displacement of long-established dietary patterns by ultra-processed foods has globally become a key driver of the escalating global burden of multiple diet-related chronic diseases, including overweight, obesity, abdominal obesity, type 2 diabetes, hypertension, dyslipidemia, cardiovascular disease or mortality, coronary heart disease or mortality, cerebrovascular disease or mortality, chronic kidney disease, Crohn’s disease, depression, and all-cause mortality [65]. A major factor leading to obesity is the imbalance between energy intake and energy expenditure [66]. It is characterized by the consumption of unhealthy and high-calorie food, including high-fat, high-sugar, or ultra-processed food. Although they have high caloric levels, these types of food provide little satiety, leading to high consumption and high fat accumulation, without the sense of fullness. The excess energy coming from a high-fat diet is stored as triglycerides in the white adipose tissue. Once overloaded, these adipose tissues become dysfunctional and lead to lipid accumulation in muscles and the liver, causing metabolic dysfunction [66]. In addition, the trend of fast food, where a high-calorie meal can be eaten in a short time before reaching the satiety limit, contributes to excessive eating and obesity [67].

A lack of physical activity is one of the major causes of obesity, and the fourth leading risk factor for mortality (http://www.who.int/data, accessed on 25 May 2026), where the body does not expend the excessive energy taken in through eating. Low physical activity not only promotes obesity but also leads to long-term metabolic dysfunction and CVD.

In addition, obesity may be hereditary due to genetic variants, where studies have shown that the genetic predisposition to obesity can interact with other environmental factors, leading to the modification of adipocytes and the body’s response to a low-calorie diet [68]. A study by Farooqi et al. estimated that up to 20% of severe obesity in childhood is due to a mutation or chromosomal abnormality [69]. A recent study on genetic subtyping of obesity shows that obesity is genetically diverse, with some variants increasing body fat while protecting against diabetes, hypertension, and heart disease. Researchers identified 205 genomic regions and eight subtypes that explain why some individuals remain metabolically healthy despite obesity. These protective effects appear early in life, highlighting the importance of genetics in shaping health outcomes and pointing toward precision medicine approaches for tailored prevention and treatment [70].

Rare monogenic variants, caused by a mutation in a single gene (i.e., MC4R mutation), are typically rare in the population but lead to early onset and severe obesity, and may be treated with targeted therapies such as melanocortin analogs [71]. However, the common polygenic variants result from many genetic variants, each with a small influence and dependent on gene–environment interaction. This type of obesity is usually identified through GWAS studies with loci like FTO and SEC16B linked to appetite and fat storage, and is harder to treat [72].

Additionally, many individuals living with obesity suffer from psycho-social problems, including issues with quality of life, social standards, and unstable mood [73]. Studies suggest that about 20% to 60% of subjects with obesity and severe obesity struggle with psychiatric problems [74]. Issues can be depression [75] with an effect on poor health-related quality of life (HRQoL) [76], eating disorders [77], anxiety [78], and substance abuse [78]. For that, visiting mental health professionals can be helpful to improve the psycho-social aspects related to obesity [73].

Moreover, studies suggest that some chemicals in the environment, called ‘obesogens’, can affect the individual’s susceptibility to obesity and lead to a significant increase in obesity prevalence [79]. In vitro, obesogens affect the differentiation of white adipose tissues, and in vivo, they can affect the storage of fats [80]. In humans, a study showed that the organotin compound tributylin (TBT), used in marine shipping and industrial water systems, has a biomagnification or bioaccumulation property and functions as a type of obesogen, which can enter the body via seafood consumption. Consequences are on adipogenesis and fat accumulation [81].

## 5. Obesity-Related Metabolic Disorders

**Figure 3:**
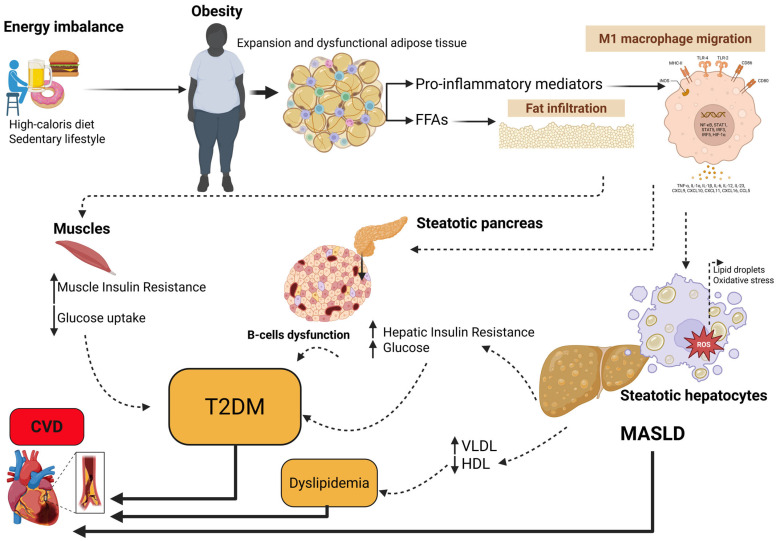
Interrelationship between Western diet, obesity, and metabolic-associated diseases. The figure illustrates the pathophysiological mechanisms linking a Western diet, characterized by high intake of fats and simple sugars (notably fructose) and low fiber content, to the development of obesity and associated metabolic disorders. Excess caloric intake leads to dysfunctional adipose tissue, which releases elevated levels of free fatty acids (FFAs) and pro-inflammatory adipokines. These factors contribute to insulin resistance in skeletal muscle and liver, impairing glucose uptake and increasing hepatic glucose production. Concurrently, the steatotic pancreas exhibits β-cell dysfunction, further exacerbating hyperglycemia and insulin resistance. Together, these changes contribute to the development of type 2 diabetes mellitus (T2DM), metabolic dysfunction-associated steatotic liver disease (MASLD), and dyslipidemia, characterized by increased very low-density lipoprotein (VLDL) and decreased high-density lipoprotein (HDL). These conditions collectively heighten the risk of cardiovascular disease (CVD). Feedback loops between these organs and metabolic pathways reinforce disease progression [11,83,84].

Excessive fat accumulation is a critical risk factor for a broad spectrum of metabolic diseases, including T2DM, metabolic dysfunction-associated steatotic liver disease (MASLD), CVD, and several other components of metabolic syndrome. This pathological process is primarily driven by an unhealthy dietary pattern rich in saturated fats and refined sugars, yet deficient in dietary fiber, which initiates a state of chronic low-grade inflammation and systemic insulin resistance. As adipose tissue becomes dysfunctional, it releases elevated levels of free fatty acids (FFAs) and pro-inflammatory adipokines, contributing to impaired insulin signaling in peripheral tissues such as skeletal muscle and liver (Figure 3) [82].

These metabolic derangements promote insulin resistance and hyperglycemia, hallmarks of T2DM. Simultaneously, ectopic fat deposition within the pancreas compromises β-cell integrity and insulin secretion, further aggravating glucose dysregulation [85].

In the liver, insulin resistance accelerates de novo lipogenesis and leads to atherogenic dyslipidemia, characterized by increased levels of very-low-density lipoprotein (VLDL) and reduced high-density lipoprotein (HDL), which substantially elevate the risk for CVD [83]. Collectively, these interrelated mechanisms highlight the systemic metabolic consequences of poor dietary habits and excessive adiposity.

### 5.1. Type 2 Diabetes Mellitus (T2DM)

The excess body fat accumulation in patients with obesity, especially visceral fat, leads to insulin resistance, where the cells become resistant and do not respond effectively to insulin [86]. To defend against this resistance, the pancreas starts to produce a higher amount of insulin to maintain normal blood glucose levels [87]. Over time, the capacity of the pancreas to maintain the normal glucose level decreases, leading to elevated blood glucose and the onset of T2DM [87]. In addition, obesity is associated with a type of insidious metabolic chronic inflammation [87], leading to an alteration of adipokine secretion and contributing further to insulin resistance and beta-cell dysfunction [88].

### 5.2. Metabolic-Associated Steatotic Liver Diseases (MASLD)

Obesity plays a significant role in liver fat accumulation, where excess fat intake accumulates in the body, including the liver, leading to liver steatosis [89]. When the capacity of adipose tissues for storing the excess fat intake is exceeded, the hepatocytes play this role and store the lipids as triglycerides [90]. If not managed, the excess fat accumulation induces chronic inflammation [91], which in turn increases the fat accumulation and damages the liver tissues, leading to MASH (metabolic-associated steatohepatitis) [10,11,92]. In another pathway, obesity is a risk factor for different metabolic syndromes, including insulin resistance and dyslipidemia, which also leads to hepatic fat accumulation [93]. In detail, insulin resistance affects the capacity of the liver to regulate lipid metabolism, leading to increased triglyceride accumulation in hepatocytes [93]. In addition, alterations in nutrient absorption and inflammatory signalling may occur due to obesity-related gut microbiota dysbiosis, leading to increased liver fat accumulation [93].

### 5.3. Cardiovascular Diseases

Obesity is a risk factor for cardiovascular diseases in many pathways and may lead to mortality. Since obesity leads to visceral fat accumulation, insulin resistance, dyslipidemia, and chronic inflammation, all these dysfunctions affect vascular functions and lead to atherosclerosis [94]. In addition, obesity increases the activity of the renin–angiotensin–aldosterone system (RAAS), which in turn increases the risk of hypertension and endothelial dysfunction, leading to an increase the cardiovascular risk [95]. Studies showed that the excessive fat accumulation also leads to an increase in total and central blood volume, a change in heart morphology and ventricular function [96]. Moreover, childhood obesity, defined through age- and sex-specific criteria due to ongoing growth and pubertal development, is a major risk factor for early-onset ASCVD [97]. Evidence from different reviews demonstrates that ASCVD originates in childhood and is significantly exacerbated by excess adiposity [98,99]. Obese children frequently exhibit metabolic risk factor clustering, including dyslipidemia, hypertension, insulin resistance, and dysglycemia, which together elevate cardiovascular risk and long-term mortality [100,101]. Moreover, childhood obesity is associated with increased odds of developing type 2 diabetes, metabolic dysfunction-associated steatohepatitis (MASH), and polycystic ovarian syndrome (PCOS) [102,103]. These metabolic disturbances often persist into adulthood, where ~50% of obese adolescents progress to severe obesity, and ~80% of those with severe obesity remain severely obese, compounding cumulative vascular damage and future disease burden [100].

### 5.4. Cholesterol Cholelithiasis

Cholesterol cholelithiasis refers to the formation of gallstones primarily composed of cholesterol, resulting from an imbalance among the three biliary lipid carriers, i.e., cholesterol, bile salts, and phospholipids within bile. If bile becomes supersaturated with cholesterol, excess cholesterol will shift from small vesicles made of Bile acid (BA)-cholesterol to larger vesicles made of large quantities of cholesterol solubilized by BA and phospholipids. Upon bile concentration in the gallbladder, vesicles tend to further aggregate and cholesterol will shift from the liquid (soluble) status to the solid status of monohydrate and then anhydrous solid cholesterol crystals which gradually aggregate into pure or mixed cholesterol stones [104,105]. Cholesterol cholelithiasis is influenced by multiple factors, including genetics, diet, metabolic disturbances, and gallbladder motility, and represents the most common type of gallstone disease [12]. Cholelithiasis affects approximately 10–20% of adults worldwide [106] with an overall prevalence of around 6% [107]. In Europe, reported rates range from 5.9% to 21.9% [108,109]. It represents one of the most frequent abdominal conditions requiring hospitalization in high-income countries [107,110]. Moreover, cholelithiasis is strongly linked to other disorders, including gallbladder cancer [111], MASLD [112], and coronary heart disease [113]. Collectively, these data emphasize the relevance of prevention and effective management strategies for cholelithiasis.

Obesity is a well-recognized risk factor that often co-occurs with cholelithiasis. Evidence indicates that elevated weight increases the likelihood of developing cholelithiasis during childhood [114,115] and adulthood [116]. Furthermore, Chen L et al. [117] demonstrated, through a Mendelian randomization (MR) analysis, that obesity raises the risk of cholelithiasis even after adjusting for confounders. Additional work also reported a positive association between rising body mass index (BMI) and cholelithiasis risk [118]. A study evaluating childhood obesity in relation to gastrointestinal (GI) disorders [119] found that genetically predicted higher childhood BMI was linked to a greater risk of cholelithiasis in adulthood. Although certain biomarkers, such as HDL cholesterol and triglycerides, have been proposed as mechanistic mediators connecting obesity to cholelithiasis [120], many molecular pathways remain undefined. Elucidating these mechanisms could provide new insights for targeted prevention and treatment of cholelithiasis later in life. A recent study showed that childhood obesity may play a developmental role in the risk of adult cholelithiasis [109]. The study showed significant genetic links between most age-specific childhood BMI stages and adult cholelithiasis. It further demonstrated that higher BMI at several early-life stages, including birth, late infancy, and early childhood, was causally associated with an increased risk of developing cholelithiasis in adulthood. In addition, the study identified MLXIPL as the strongest shared molecular signal connecting childhood BMI to adult cholelithiasis, suggesting a potential biological pathway underlying this relationship [109].

## 6. Obesity in Young People

Approximately 30% of children and adolescents in the US live with overweight or obesity [121]. Today, the global burden of overweight and obesity affects more than 245 million children aged between 5 and 14 years, 249 rates are projected to rise significantly by 2050. Alarmingly, younger individuals face a future of obesity-related health complications that will place increasing pressure on healthcare systems, with costs expected to reach approximately US$4.32 trillion by 2035 [122]. Childhood obesity is particularly concerning due to the vulnerability of this age group and the early onset of related health issues [123]. Metabolic disturbances such as oxidative stress, energy imbalance, and genetic alterations can appear even before puberty, increasing the risk of long-term health problems [124]. Since many obesity-related factors are established early in life, investing in health from preconception to adolescence is not only effective but also highly cost-efficient, with potential returns of up to ten times the investment.

The causes behind the surge in youth obesity are multifaceted, stemming from a combination of biological, economic, and environmental influences [125]. As for adults, the development of obesity in youth subjects is influenced by multiple factors that interact throughout the lifespan, including individual factors such as epigenetics, biology, psychology, and specific behaviours such as nutrition, low or no physical activity, and sleep disorders. Genetics/epigenetics play a role, but they are often amplified by unhealthy eating patterns and socioeconomic circumstances [126]. As underscored by a recent action in The Lancet, the global proliferation of ultra-processed foods has become one of the most urgent yet inadequately addressed threats to human health in the 21st century [127]. Indeed, the global proliferation of ultra-processed foods poses a significant threat to children’s health, and this aspect requires urgent action from governments to protect children’s rights to adequate nutrition and health [128]. Affordable, calorie-dense, and sugar-laden ultra-processed foods are more accessible than nutritious options, particularly for low-income families who may also lack time for meal preparation [129,130], and children are highly vulnerable in terms of malnutrition, metabolic alterations, and mental health concerns related to ultra-processed foods, which deliver abnormally high levels of sweet, salty, and artificially flavoured foods [131]. Larger meal portion sizes served at restaurants may significantly increase children’s caloric intake by ≈ 86 kcal per meal, and if repeated, this exposure can override the normal satiety cues and contribute to long-term obesity [132].

Excessive sedentary screen time, especially TV viewing and mobile device use, is strongly linked to higher obesity risk in children and adolescents, due to low physical activity and unhealthy snacking habits [133].

Despite progress in identifying individual risk elements, the exact mechanisms through which these factors contribute to obesity remain unclear. Individual factors interact with the environment, including exposure to chemicals. Commercial determinants, particularly industry strategies that promote certain food products and ideas, can also help to shape the environment. Social determinants and policies affect many aspects of the lives of people living with obesity. Obesity in young individuals is also associated with psychological and neurological disorders, creating bidirectional relationships in which psychiatric conditions increase the risk of obesity and obesity can trigger disorders through immune, metabolic, and endocrine alterations. In addition, obesity promotes an increased risk of cardiovascular and metabolic disease through insulin resistance and various types of cancer [134,135]. Strong evidence suggests that comprehensive food environment policies can contribute to reducing childhood obesity [136]. A notable example is Chile’s 2016 Food Labelling and Advertising Law (FLAL), which combined mandatory front-of-package black warning labels with restrictions on food marketing to children and the sale of unhealthy foods in schools [137]. Recently, in a large national cohort study involving more than 321,000 children aged 4–6 years, exposure to the first phase of the policy was associated with a significant reduction in the likelihood of excess weight (overweight or obesity) [138]. The greatest effect was observed among children exposed to the policy for 18 months, with the probability of excess weight decreasing by 2.85% in girls and 2.40% in boys, while even 6 months of exposure produced significant reductions. These findings provide some of the strongest causal evidence to date that comprehensive regulatory policies targeting the food environment can effectively reduce excess weight in early childhood, supporting their adoption as scalable public health strategies to address the growing childhood obesity epidemic [138].

## 7. Advancing Obesity Research and Redefining Diagnostic Criteria

Despite extensive research into the pathophysiology of obesity, significant challenges persist in the areas of diagnosis, epidemiological assessment, clinical heterogeneity, and policy implementation [139]. In addition, no obvious categories for the anthropometric variables exist, and it is not clear what the levels of the anthropometric criteria are based on [140]. Recognizing these limitations, a growing number of international initiatives are redefining how obesity is understood and managed to develop more precise, equitable, and effective interventions.

**Figure 4:**
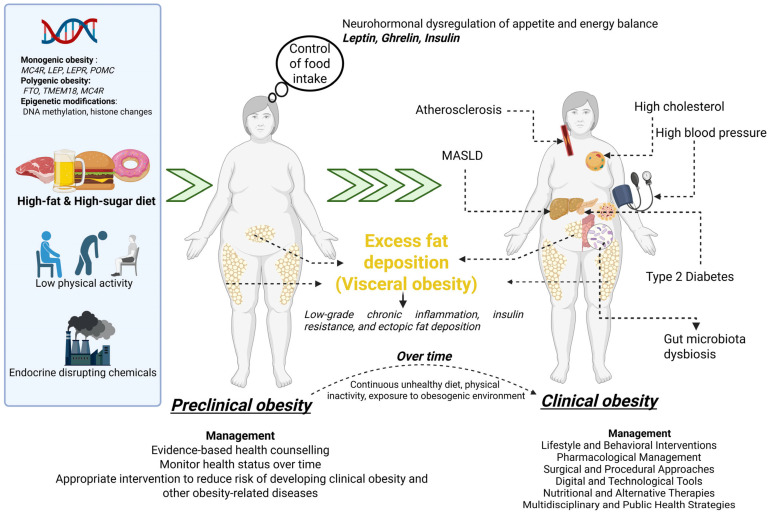
Difference between “preclinical obesity” and “clinical obesity” [55]. Preclinical obesity is a multifactorial case caused by genetic, epigenetic, environmental, and behavioral risk factors that contribute to neurohormonal dysregulation (leptin, ghrelin, insulin), promoting visceral fat accumulation and insulin resistance. It could be managed by controlling food intake, health consultation, monitoring health status over time, and early intervention to prevent its transition to clinical obesity [141]. As obesity progresses, chronic inflammation and ectopic fat deposition exacerbate cardiometabolic risks, including T2DM, MASLD, dyslipidemia, hypertension, and atherosclerosis [142]. Gut microbiota dysbiosis further disrupts metabolic homeostasis [143,144]. Management of clinical obesity includes evidence-based interventions ranging from lifestyle modification to pharmacological therapy, bariatric procedures, digital health tools, and public health strategies.

A landmark contribution in this regard comes from the 2025 Commission Report, which proposes a paradigm shift in the diagnostic criteria for obesity. Developed in collaboration with 56 international experts, the Commission introduces a novel distinction between “clinical obesity”, i.e., a high BMI confirmed by measures of body fat, if available, or by at least 1 anthropometric criterion (which includes waist circumference, waist-to-hip ratio, or waist-to-height ratio), and “preclinical obesity” in people without a high BMI and at least 2 anthropometric criteria [55]. This evidence-based framework seeks to move beyond the traditional reliance on body mass index (BMI) as a singular diagnostic tool, acknowledging its limited capacity to capture the biological and clinical diversity of individuals affected by excess adiposity. The new classification defines preclinical obesity as a state of excess fat accumulation without current organ dysfunction or life impairment but with a high risk of future health consequences, while clinical obesity is recognized as a chronic, systemic disease characterized by pathological fat accumulation with demonstrable physiological harm (Figure 4). The EASO, which published its own obesity “framework” in July 2024, considered that the Lancet Commission report could give rise to problematic concepts that could delay early prevention of obesity comorbidities. To introduce the concept of a “preclinical obesity” category might be responsible for a watchful waiting strategy that could delay interventions and dangerously worsen long-term health outcomes.

The necessity of capturing this preclinical risk is underscored by epidemiological data that show that the BMI cutoffs obscure hidden CVD vulnerabilities. A recent study among young women found a low risk of several CVDs with a low-normal BMI (about 22 kg/m^2^); however, an increased risk in the highest BMI among the normal range (between 22 and 25 kg/m^2^) has been detected [145]. Consequently, while the Commission underscores the need for practical and globally applicable diagnostic tools, it also calls for a revision of current treatment eligibility criteria and a greater emphasis on early intervention and prevention in those with preclinical risk.

These developments are aligned with and further informed by the recent consensus statement from the European Society for Clinical Investigation (https://esci.eu.com/), which identifies critical knowledge gaps and proposes strategic directions for future obesity research and policy [11]. Chief among the concerns are the inadequacy of BMI-centric epidemiological models, the lack of standardized long-term obesity surveillance, and a deficient understanding of phenotypic variability in obesity-related cardiometabolic risk. Additionally, the socio-economic and environmental drivers of obesity and their role in shaping transitions across phenotypes remain insufficiently elucidated [11]. The consensus report advocates for an integrated, syndemic framework, reflecting the interconnected biological, societal, and ecological determinants of obesity. This approach supports the development of tailored, community-level interventions embedded within global health strategies. The use of emerging technologies, including artificial intelligence, is emphasized to refine predictive models, enhance data resolution, and better stratify risk across diverse populations [11]. Taken together, the advancement of these consensuses highlights the need for harmonization of anthropometric and biochemical markers, inclusive public health messaging, and efforts to counteract stigma in clinical and societal discourse. Notably, they stress that the historical one-size-fits-all approach to obesity care is no longer tenable. Instead, there is an urgent need for precision medicine models, grounded in individualized assessment and supported by evidence-based public policies that reflect the true complexity of obesity as a disease and societal challenge [146]. With this idea in mind, Fourman et al. [147] applied the new Lancet 2025 criteria to the “All of Us” cohort, reporting that when BMI is high, most participants met at least one anthropometric criterion as well. By contrast, many other participants who did not meet the BMI criteria did meet the anthropometric criteria for obesity. Such methodological approaches will be required in the future, since the adoption of a new definition of obesity that incorporates anthropometric measures can significantly increase the prevalence of obesity and has major implications for clinical practice and public policy. In addition, further studies are warranted looking at the outcomes when stratifying individuals at high risk of organ dysfunction and long-term complications with anthropometric-only obesity and preclinical obesity as distinct entities [147].

The 2025 Lancet Commission also reframes pediatric obesity as a heterogeneous chronic disease that extends beyond excess body weight and emphasizes the distinction between preclinical obesity and clinical obesity [55]. In children and adolescents, BMI-for-age percentiles remain an appropriate screening tool [148], but they should not be used in isolation to establish the diagnosis. Rather, obesity should be confirmed by evidence of excess adiposity using anthropometric measures (e.g., waist circumference or waist-to-height ratio), skinfold thickness, or direct body composition assessment when available [149], together with a comprehensive clinical evaluation to identify obesity-related organ dysfunction and functional impairment. Children with preclinical obesity exhibit excess adiposity without established clinical consequences but remain at increased risk for progression, warranting early preventive interventions and longitudinal monitoring. By contrast, clinical obesity is defined by the presence of obesity-related organ dysfunction or clinically significant impairment affecting one or more organ systems, including metabolic, cardiovascular, respiratory, musculoskeletal, hepatic, reproductive, renal, neurological, or psychosocial domains [55]. This paradigm recognizes that obesity-related complications often begin during childhood, although many initially manifest as subclinical abnormalities because of shorter disease duration and greater physiological compensatory capacity. Consequently, early identification and timely intervention are essential to prevent progression toward irreversible organ damage and adult cardiometabolic disease. This approach also aligns with the 2023 American Academy of Pediatrics Clinical Practice Guideline, which advocates early, intensive, family-centered treatment using a continuum of evidence-based interventions—including health behavior and lifestyle treatment, pharmacotherapy, and metabolic bariatric surgery for appropriately selected adolescents—rather than the traditional stepwise escalation based solely on BMI severity [148].

## 8. Strategies for Losing Weight in Adults

Managing excess adiposity requires a structured clinical approach that shifts focus from simple BMI metrics to a comprehensive evaluation of adipose-tissue function and organ-specific damage. In strict alignment with the 2025 Lancet Diabetes and Endocrinology Commission guidelines, therapeutic strategies are divided into two distinct tiers: preclinical obesity interventions focused on non-pharmacological risk mitigation for patients without functional organ impairment, and clinical obesity interventions, which require medical and surgical escalation for patients with established, adipose-driven comorbidities.

**Figure 5:**
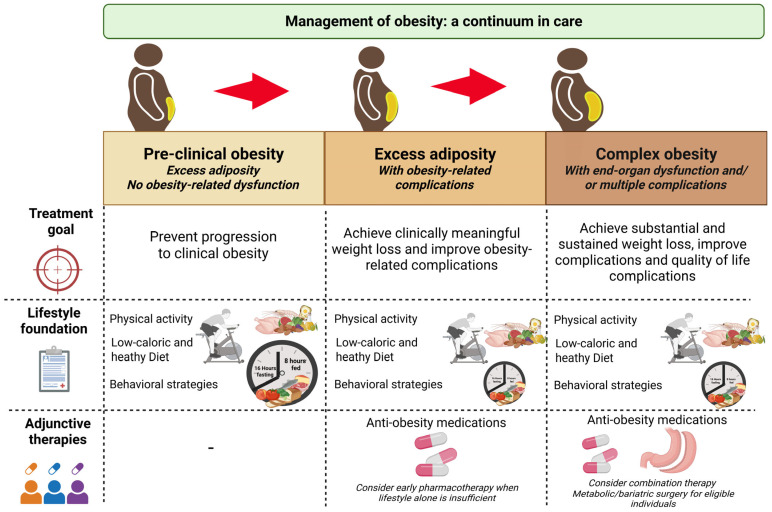
Updated framework for the management of obesity according to the 2025 Lancet Commission [55]. Management of obesity is presented as a continuum of care based on the 2025 Lancet Commission definition of obesity, which distinguishes preclinical obesity (excess adiposity without obesity-related organ dysfunction or functional limitations) from clinical obesity (excess adiposity associated with objective signs and/or symptoms of organ dysfunction, reduced functional capacity, or obesity-related disease). Unlike traditional BMI-based classifications, treatment intensity is guided by the presence and severity of obesity-related clinical consequences rather than BMI alone. Lifestyle interventions, including healthy dietary patterns, increased physical activity, and behavioral support, remain the cornerstone of management across the continuum. Anti-obesity pharmacotherapy should be considered for individuals with clinical obesity when lifestyle interventions alone are insufficient, while metabolic/bariatric surgery is recommended for eligible patients with advanced disease or severe obesity-related complications. BMI remains a useful screening tool but should not be used as the sole criterion for diagnosing or staging obesity.

Comprehensive evidence-based obesity treatment combines behavioral interventions, lifestyle, pharmacological, and surgical approaches tailored to individual patient phenotypes (Figure 5) [11,55,150]. Rather than deploying these interventions as a sequential menu based on body mass index (BMI) thresholds, the 2025 Lancet Commission diagnostic paradigm mandates mapping strategies directly to the patient’s phenotypic staging. Preclinical obesity focuses on non-pharmacological risk containment, while clinical obesity requires immediate medical and procedural escalation to halt cumulative organ damage [151].

### 8.1. Interventions for Preclinical Obesity

To transition from a conceptual framework to targeted therapeutic implementation, non-pharmacological strategies within the preclinical tier must be personalized according to patient compliance, baseline metabolic profiles, and target risk factor reduction. A comprehensive phenotypic overview of these primary behavioral, dietary, and lifestyle interventions is summarized in Table 2.

#### 8.1.1. Physical Activity

As defined, physical activity describes any skeletal muscle movement that leads to energy expenditure (WHO). In addition to its efficient effect on weight loss, physical activity improves metabolic disorders such as type 2 diabetes, reduces stress and anxiety, and decreases the risk of heart disease and certain tumors (https://www.who.int/news-room/fact-sheets/detail/physical-activity, accessed on 25 May 2025). Regarding physical activity’s beneficial healthy effect, studies have shown that aerobic training has a greater effect on weight loss than resistance training [179], since the expenditure of energy during aerobic exercise is higher than the expenditure during resistance training [180]. Combined with a healthy diet, physical activity showed a higher effectiveness in reducing body weight and improving body composition [181]. Different types of physical activity exist, but aerobic and high-intensity interval training are recommended for weight loss in subjects with obesity [182], with duration and intensity depending on the weight and the goal [183].

#### 8.1.2. Dietary Approaches and Macronutrient Patterns

Dietary changes for weight loss within the preclinical tier focus on altering systemic energy balance while improving long-term nutritional quality [184]. Selecting a specific dietary approach depends on the metabolic profile of the patient, behavioral compliance capacity, and target risk factor reduction [185].

##### Ketogenic Diet

The ketogenic diet is one of the most popular diets in the last few years, consisting of very low carbohydrate consumption with varying levels of fat and protein [186]. When carbohydrate intake is restricted, hepatic gluconeogenesis maintains the glucose supply by producing non-carbohydrate sources such as pyruvate, lactate, and glycerol. When glucose storage is depleted, ketone bodies are produced as an alternative fuel. So the hormonal pivot here is the decreased insulin secretion that stops glycogen and fat storage while activating lipolysis and fatty acid oxidation [187]. Previous studies showed its effect in reducing fat mass and visceral fat mass, while preserving the lean mass in subjects [158]. The ketogenic diet has been shown to have beneficial effects on the blood lipid profile, in addition to an anti-inflammatory and cardioprotective role due to the state of ketosis, the decrease in simple sugar, and the higher omega-3 fatty acid consumption [188]. A meta-analysis was conducted to assess the effect of the ketogenic diet on weight loss and glycemic profile in overweight diabetic patients, which has shown a beneficial effect on weight loss, decreasing body circumference, glycated hemoglobin, triglycerides, and increasing high-density lipoproteins [189].

##### Very Low-Calorie Diet (VLCD)

The very low-calorie diet is considered a strategy for rapid weight loss that showed a long-term advantage by maintaining the loss of weight for over 36 months [190]. Subjects practicing this type of diet lost a high percentage of their weight, divided into fat mass and lean mass, which makes it not the best way to improve body composition and maintain the weight loss [191]. While old literature showed that a VLCD inducing rapid weight loss, i.e., >1.5 kg per week, becomes a risk factor for the formation of cholesterol gallstones in up to 30% of individuals due to cholesterol supersaturation in bile. Modern VLCD formulations incorporating supplemental dietary fiber and bile acid binders have significantly decreased the risk of clinical gallstones to below 10% [106,192].

Moreover, studies show that only a small fraction of patients maintain significant weight loss after long-term follow-up, while new cases of vascular events and type 2 diabetes have been reported after initial success [193]. Some patients showed persistent DNA damage, suggesting genomic instability risks [194]. Importantly, from a pediatric safety perspective, VLCDs require medical supervision due to recent clinical trial data that demonstrated that up to %95 of developing youth experience acute adverse events, including negative nitrogen balance and severe metabolic fatigue, during intensive energy restriction, mandating that VLCD utilization remain restricted to a highly monitored, specialized multi-disciplinary clinical setting [195].

##### High-Protein Diet

A high-protein diet is used to utilize fat-free skeletal mass during the energy restriction periods. Mechanistically speaking, elevated amino acid availability will enhance the mammalian target of rapamycin complex 1 pathway, stimulating muscle protein synthesis and enhancing the satiety feeling through the modulation of anorexigenic signaling peptides. In such a caloric-deficit diet, clinical studies showed that consumption of more than 2 g of protein per gram per day is required for maintaining the lean body mass within a calorie-deficient diet [160]. Additionally, A recent study on 80 subjects with obesity or overweight has shown a significant increase in microbial alpha-diversity and composition after the high-protein diet [196].

##### Low-Fat Diet

Low-fat strategies leverage the high energy density of dietary lipids relative to hydrophilic macronutrients to induce a passive energy deficit. Clinical studies have shown the effect of high-fat diets and the consumption of junk food high in saturated fatty acids on the alteration of the gut microbiota and its association with obesity-related metabolic diseases. A low-fat diet seems to be a healthy option for weight loss and the prevention of its consequences [197].

##### Mediterranean Diet (MD)

**Figure 6:**
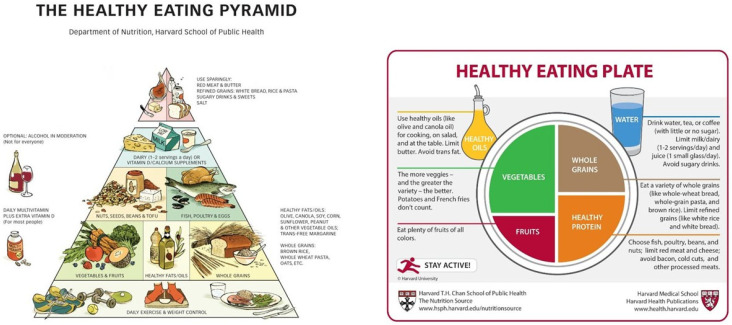
The Healthy Eating Pyramid addresses other aspects of a healthy lifestyle—exercise, weight control, vitamin D and multivitamin supplements, and moderation in alcohol for people who drink—so it is a useful tool for health professionals and health educators [199]. Copyright © 2008. For more information about The Healthy Eating Pyramid, please see The Nutrition Source, Department of Nutrition, Harvard T.H. Chan School of Public Health, www.thenutritionsource.org (accessed on 6 April 2026), and Eat, Drink, and Be Healthy. Free Press/Simon & Schuster Inc. The healthy eating plate is a guide for creating healthy, balanced meals, whether served at the table or packed in a lunch box. Copyright © 2011, Harvard University. For more information about The Healthy Eating Plate, please see The Nutrition Source, Department of Nutrition, Harvard T.H. Chan School of Public Health, www.thenutritionsource.org (accessed on 6 April 2026), and Harvard Health Publications, www.health.harvard.edu (accessed on 6 April 2026).

The concept of the MD emphasizes plant-based foods and healthy fats, where the main components are vegetables, fruits, whole grains, fish, and extra virgin olive oil, which is the primary source of additional fat, with low-alcohol intake allowed [198]. A popular graphical presentation of the MD dietary pyramid (Figure 6) represents the main components of MD, together with their relatively recommended quantity of consumption related to daily/weekly intake. Further elaboration provides the figure of the healthy eating plate, a guide for creating healthy, balanced meals, whether served at the table or packed in a lunch box [199,200]. Both cartoons point to the beneficial role of daily exercise and staying active (see below). Accumulating evidence from recent systematic reviews and meta-analyses supports the MD as an effective dietary strategy for weight management, although its weight-loss efficacy is generally comparable to other evidence-based dietary approaches. A network meta-analysis of RCTs in overweight and obese adults demonstrated that the MD consistently promoted clinically meaningful weight loss, although low-carbohydrate diets achieved a greater reduction in body weight (mean difference: −2.70 kg versus the MD) [163]. Similarly, in adults with T2DM and overweight or obesity, the MD ranked highest for improving glycemic control, while differences in weight loss between dietary patterns were small and of limited clinical relevance, suggesting that adherence and metabolic benefits may outweigh modest differences in weight reduction [165]. Furthermore, a recent meta-analysis in patients with MASLD showed that the MD significantly reduced body weight (weighted mean difference: −2.38 kg), body mass index (−0.70 kg/m^2^), and waist circumference (−1.56 cm) compared with control interventions [166].

Among the various dietary approaches, MD has the strongest evidence for improving long-term health outcomes. A systematic review and network meta-analysis of 40 randomized controlled trials involving 35,548 participants at increased cardiovascular risk found that Mediterranean dietary programs significantly reduced all-cause mortality (odds ratio [OR] 0.72, 95% CI 0.56–0.92), cardiovascular mortality (OR 0.55, 95% CI 0.39–0.78), stroke (OR 0.65, 95% CI 0.46–0.93), and non-fatal myocardial infarction (OR 0.48, 95% CI 0.36–0.65) [164]. Collectively, these findings indicate that while the Mediterranean diet may not consistently produce the greatest absolute weight loss, it achieves sustained reductions in adiposity alongside broad cardiometabolic and hepatic benefits, making it one of the most comprehensive dietary strategies for the prevention and management of obesity.

#### 8.1.3. Fasting Regimens

Intermittent fasting (IF) is an alternation between eating times and fasting times [167]. Research indicates that intermittent fasting can offer numerous health benefits, including weight loss, improved insulin sensitivity, and reduced risk of type 2 diabetes [168]. Additionally, intermittent fasting may enhance heart health by lowering blood pressure and cholesterol levels [169]. It also promotes cellular repair processes and may reduce inflammation and oxidative stress [170]. Several types of intermittent fasting have been studied, which alternate between periods of fasting and eating [201].

##### Alternate-Day Fasting (ADF)

This method involves alternating between days of no caloric intake and days of unrestricted eating. Modified versions allow for a small caloric intake (up to 600 kcal) on fasting days [171]. A systematic review and meta-analysis of randomized controlled trials found that ADF significantly reduces body weight, body mass index (BMI), total cholesterol, low-density lipoprotein (LDL), triglycerides, and blood pressure [202].

##### Time-Restricted Eating (TRE)

This approach limits the daily eating window to a specific number of hours, typically ranging from 4 to 12 h, with the remaining hours spent fasting. A previous study found that early TRE (eating within a 6 h window starting in the morning) was more effective for weight loss and fat loss compared to a control eating schedule [173]. Additionally, TRE has been shown to improve cardiovascular health markers, such as lowering blood pressure and cholesterol levels [174]. However, the long-term sustainability and potential risks of TRE, particularly in different populations, require further investigation [175].

##### The 16:8 Method

The 16:8 intermittent fasting regimen, where individuals fast for 16 h and eat during an 8 h window, has garnered significant attention for its potential health benefits. Research indicates that this eating pattern can lead to weight loss, improved metabolic health, and enhanced insulin sensitivity [203]. Intermittent fasting has been shown to decrease levels of insulin and improve insulin sensitivity, which can be beneficial for individuals with or at risk of type 2 diabetes [204]. Moreover, intermittent fasting could improve circadian rhythms and reduce the risk of chronic diseases such as cardiovascular disease. These findings suggest that the 16:8 intermittent fasting method may offer a sustainable and effective approach to improving overall health and preventing metabolic disorders [205].

##### Ramadan IF

Intermittent fasting is one of the religious practices in the Islamic population called ‘’Ramadan intermittent fasting’’, where healthy adults practice fasting from food and drinks, including water, from dawn to sunset for 30 days per year [206]. Depending on the geographical location, fasting hours can vary between 12 and 22 h (mean 12–14 h) [207]. However, fasting is not required for premature children, the elderly, the sick, and pregnant and lactating women [208]. Studies showed that Ramadan intermittent fasting reduces body weight and body fat mass, improves metabolic syndrome, decreases waist circumference, fasting serum glucose, serum lipid profile, insulin sensitivity, and improves GI symptoms and motility [206]. In addition, Ramadan intermittent fasting was able to reduce subcutaneous and visceral fat and improve liver steatosis [209]. Hormonal changes, such as alterations in leptin and adiponectin levels, which regulate appetite and satiety, have also been observed [210].

#### 8.1.4. Natural Compounds, Functional Foods, and Nutraceuticals

Beyond standardized macronutrient diets, plant-derived bioactive extracts represent a rapidly expanding area of adjunctive research. Under the 2025 Lancet Commission staging framework, these natural elements are classified strictly as experimental strategies for preclinical obesity management to assist with early metabolic risk containment. Crucially, none of the natural compounds, functional foods, or nutraceutical extracts detailed in this review possess regulatory approval from major bodies (such as the FDA or EMA) for the treatment of overweight or obesity, nor have any completed robust Phase III clinical testing.

Preclinical in vitro and in vivo models (summarized in Table 3) provide substantial mechanistic insight. These studies demonstrate that major phytochemical classes, including polyphenols, alkaloids, saponins, and terpenes, target pathways by downregulating adipogenic transcription factors (such as PPARγ and C/EBPα), inhibiting pancreatic lipase, and activating cellular energy sensors like AMPK and SIRT1.

Polyphenols represent one of the most extensively studied phytochemical classes in obesity research [222], owing to their antioxidant, anti-inflammatory, and metabolic regulatory properties [223]. Preclinical studies indicate that polyphenols exert their anti-obesity effects primarily through modulation of insulin signaling, inhibition of adipogenic transcription factors, and activation of AMPK-dependent pathways [211,212,213,214].

Alkaloids have also shown promising anti-obesity effects, particularly through inhibition of digestive enzymes and suppression of adipocyte differentiation [215,216,217].

Saponins exert anti-obesity effects through the regulation of lipid metabolism [218] and glucose homeostasis [219]. Terpenes contribute to obesity prevention primarily through antioxidant, anti-inflammatory, and lipid-lowering mechanisms [220,221].

To transition from a theoretical framework to actionable clinical guidance, multidisciplinary care coordination must follow strict, trigger-based operational guidelines. Primary care professionals are the center for all obesity treatment, and the endocrinologist is the specialist of reference for the therapeutic approach to this condition in high-risk patients or after the failure of primary care interventions [224].

Table 4 provides recent clinical evidence that natural compounds, functional foods, and nutraceuticals may exert beneficial effects on obesity and obesity-related metabolic disturbances, including insulin resistance, dyslipidemia, hepatic steatosis, and adipokine dysregulation. Most of the available evidence derives from randomized, double-blind, placebo-controlled trials conducted between 2023 and 2025, with intervention durations ranging from acute postprandial assessments to long-term supplementation up to 12 months.

Across multiple trials, reductions in body weight, body BMI, and adiposity-related parameters emerge as the most consistent outcomes. Significant decreases in body weight and BMI were reported following supplementation with curcumin [228], licorice extract combined with energy restriction [229], white kidney bean extract [231], fiber blends containing glucomannan, inulin, and psyllium [232], green coffee bean extract [225], Cyperus rotundus extract [240], sunflower seed extract [227], and *Nitraria retusa* extract [233]. Several interventions also demonstrated preferential reductions in visceral or abdominal fat, as observed with Camu Camu extract [230], Oligonol^®^ [238], and combined Qingyanyin granules with press needle therapy [241], highlighting potential benefits for cardiometabolic risk reduction beyond total weight loss.

Improvements in glucose homeostasis and insulin sensitivity and favorable changes in lipid metabolism represent another recurring finding. While heterogeneity in study design, duration, and endpoints remains, these findings support the continued investigation of nutraceutical-based strategies as adjunctive, generally safe approaches for the prevention and management of obesity and its related metabolic disorders. However, the current evidence remains predominantly preclinical. Furthermore, nutraceuticals are not approved as drugs for obesity treatment by major regulatory agencies, and no clinical guidelines currently recommend their routine use for obesity management due to the limited availability of robust long-term clinical evidence.

### 8.2. Interventions for Clinical Obesity

When patients present with clinical obesity, lifestyle and nutritional recommendations are insufficient at this stage; management must be more advanced. This clinical tier mandates the integration of anti-obesity pharmacotherapies or bariatric surgery to restore metabolic homeostasis, alleviate systemic inflammation, and prevent long-term cardiovascular and hepatic mortality. The mechanistic foundations, clinical advantages, and primary limitations of current regulatory-approved anti-obesity medications and bariatric surgeries are detailed in Table 5.

#### 8.2.1. Antiobesity Drugs

After the failure of the behavioural intervention, lifestyle changes, and maintaining weight loss, the use of anti-obesity drugs becomes a choice for weight management. The goal of these drugs is to reduce weight, maintain weight loss, and improve health status. The use of pharmacological therapy for weight loss is usually recommended for individuals with clinical obesity with weight-related comorbidities, or central adiposity (e.g., elevated waist circumference) with weight-related comorbidities [21,262]. Access to drugs is also possible for individuals without the above-mentioned conditions, but this is raising concern due to the economic implications of obesity treatments [263]. The choice of an anti-obesity drug depends on medication efficacy and adverse effects. Additional issues include patient contraindications, comorbidities, preferences, and out-of-pocket costs (Table 6).

Recent advancements in pharmacotherapy for body weight management have introduced several classes of medications, each targeting overweight and obesity management through distinct mechanisms. The GLP-1-based therapies are considered the first-line anti-obesity medication, but in case of intolerance, alternatives such as central nervous system (CNS) stimulants/appetite suppressants or lipase inhibitors may be used [264].

##### GLP-1-Based Therapy the GLP-1 (Glucagon-like Peptide-1)-Based Drugs

They were first used as anti- diabetic medication. However, the observation of 2% to 5% of body weight during the treatment highlighted the hypothesis of their effect on weight loss and obesity management [265]. They act through multiple mechanisms, where they reduce appetite, leading to decreased food intake, and they slow the rate at which the stomach empties, which prolongs feelings of fullness. Additionally, they enhance insulin secretion in response to meals and reduce glucagon release, helping regulate blood glucose levels [266]. Moreover, these drugs have shown beneficial metabolic effects by improving glycaemic control, reducing cardiovascular risk, and potentially reducing liver fat and sleep apnea severity [265]. A clinical study by Zander et al. has shown a significant weight loss (for a period of 6 weeks), an increase in satiety and fullness feeling, and a reduction in food intake after a continuous GLP-1 infusion in T2DM patients [267].

The cardiovascular efficacy of long-term GLP-1 receptor agonist therapy in individuals with preclinical or clinical obesity (independent of a diabetes diagnosis) was established by the landmark SELECT (Semaglutide Effects on Heart Disease and Stroke in patients with overweight or obesity) trial. The SELECT trial was performed as a randomised, double-blind, multicentre, placebo-controlled, event-driven phase 3 trial in 41 countries, which evaluated the effect of semaglutide on patients with or without heart failure at enrolment. Treatment with 2.4 mg semaglutide resulted in MACE reduction and composite heart failure compared to placebo [268]. Another multicentre, double-blind, randomized, placebo-controlled, event-driven superiority trial was conducted on 17,604 patients with established atherosclerotic cardiovascular disease and clinical obesity, where 8803 received 2.4 mg semaglutide for approximately 40 months, showing a statistically superior 20% relative risk reduction in MACE in the semaglutide group [269]. Taken together, these landmark data demonstrate that semaglutide 2.4 mg offers robust, systemic cardioprotection and MACE reduction in patients with obesity independent of glycemic status or baseline heart failure.

The most recently used GLP-1-based drug is tirzepatide, which plays a role in the regulation of appetite and insulin secretion and is an agonist of glucose-dependent insulinotropic polypeptide (GIP) by enhancing insulin release and improving fat metabolism [242]. It was primarily used as a medication for T2DM, where it helps improve blood sugar control by stimulating insulin release and reducing glucose production [243]. Studies have shown its ability to induce a dose-dependent bodyweight loss ranging from 7·0 to 9·5 kg [244].

Beyond dual-agonist therapies, there is a triple-hormone receptor agonist targeting the GLP-1, GIP, and glucagon (GCG) receptors. Retatrutide (LY3437943) represents this vanguard, where there is an integration of glucagon receptor agonism that enhances energy expenditure through increasing both thermogenesis and the metabolic activity of the liver, balancing the drop in resting metabolic rate typically seen with weight reduction. Phase 2 clinical data were so promising, and Phase 3 trials are currently underway to evaluate the long-term systemic durability. A phase 2a trial performed in 2024 on 98 MASLD participants showed that normal liver fat (<5%) was achieved after 24 weeks in more than 85% of participants in the two highest dose groups (79% (8 mg), 86% (12 mg)), suggesting it is an effective therapeutic agent for MASLD treatment [270].

However, emerging real-world evidence and clinical extension trials underline that weight reduction achieved via GLP-1 receptor agonists is dependent on the continuity of the treatment, with weight rebound occurring after discontinuation. In a STEP 1 randomized trial, 1961 adults with clinical obesity and without diabetes were taking 2.4 mg semaglutide for 68 weeks, and then it was discontinued for one year; participants regained two-thirds of their prior weight loss with similar changes in cardiometabolic variables [271]. Similarly, the SURMOUNT-4 trial showed a weight loss of 20.9% after a 36-week tirzepatide treatment, and after discontinuation, there was 14.8% of weight regain over one year [243]. These findings confirm that obesity operates as a chronic, relapsing metabolic disease, illustrating that pharmacological GLP-1 inhibition must be viewed as a long-term maintenance strategy rather than a temporary intervention.

Alternatively, semaglutide 2.4 mg and liraglutide 3.0 mg are subcutaneously used as abdominal injections, and they have also shown their efficacy in weight loss [151]. Some studies have shown the effects of semaglutide on glycemia and lipid improvement, as well as a cardiovascular beneficial effect, in addition to its anti-obesity management effect [246,247]. As of December 2025, the WHO has released new guidelines addressing the use of GLP-1-based therapies for the treatment of obesity, which should be used long-term and in combination with intensive behavioral therapy to maximize and sustain weight loss benefits [272]. An unmet need with these drugs is equitable access to therapy [263], and this aspect is also discussed in the guidelines [272].

A further therapeutic pharmacological approach in obesity is the combination therapy targeting GLP-1 along with other GI hormones. Cagrilintide is a long-acting amylin analogue that helps regulate appetite. Amylin is a 37-residue peptide hormone that is co-secreted from the pancreatic β-cells with insulin (insulin: amylin ratio 100:1). Amylin is therefore involved in glycemic regulation by slowing gastric emptying and promoting satiety. This effect prevents post-prandial glycemic spikes [273]. In a recent randomized trial based on 3417 patients with clinical obesity, combination treatment with cagrilintide 2.4 mg and semaglutide 2.4 mg led to greater weight loss compared with either agent alone or placebo after 68 weeks [274]. The study remains investigational, but is still promising for the future.

Despite their proven clinical efficacy, the widespread implementation of GLP-1 receptor agonists is constrained by major economic and healthcare system barriers. A recent lifetime cost-effectiveness analysis estimated that although tirzepatide and semaglutide improve health outcomes by approximately 0.35 and 0.25 quality-adjusted life years (QALYs), respectively, neither therapy is cost-effective at current US prices; semaglutide would require an estimated 81.9% price reduction and tirzepatide a 30.5% reduction to meet conventional willingness-to-pay thresholds [275]. From a payer perspective, the Institute for Clinical and Economic Review projected that expanding Medicare coverage for obesity pharmacotherapy would increase spending by US$24.9 billion over 10 years, with only modest early healthcare savings, highlighting the tension between long-term value and short-term affordability [276]. These financial and reimbursement barriers contribute to substantial socioeconomic and international disparities in access, underscoring the need for pricing reforms and equitable reimbursement policies to maximize the population-level benefits of these therapies [277].

##### CNS Stimulants/Appetite Suppressants

For patients with intolerance to GLP-1-based therapy, or those who prefer oral administration, other choices like CNS stimulants and an appetite suppressant combination may be used. Appetite Suppressants primarily act centrally on brain pathways to reduce food intake; long-term use must be carefully monitored.

The combination of phentermine–topiramate has shown a significant effect on body weight loss during the first years of use [248]. When taken together, phentermine and topiramate act synergistically, producing more effective appetite suppression and reductions in caloric intake compared to either drug alone [249]. However, the phentermine/topiramate combination drug was only US-approved but has only gained limited approval in Europe in 2024 [278]. Naltrexone–bupropion is also a reasonable alternative combination that can be used for long-term use; however, it has lower efficacy compared with phentermine–topiramate and GLP-1 receptor agonists [279].

##### Lipase Inhibitors

Synthetic inhibitors of pancreatic lipase are considered very effective for weight management, since they play a role in the hydrolysis of triglycerides into monoglycerides and free fatty acids. These free fatty acids get stored in adipose tissues, leading to weight gain [280]. Lipase inhibitors act locally in the GI tract without affecting the central nervous system or insulin secretion, suitable for patients who prefer non-hormonal weight loss strategies. They alter fat digestion by inhibiting pancreatic lipases. In addition, they increase fecal fat excretion because of incomplete fat hydrolysis and absorption. In normal individuals with a diet that contains 30% fat, lipase inhibitors, such as orlistat, lead to a dose-dependent increase in fat excretion in stool, which decreases the absorption of approximately 25 to 30% of calories coming as fat [251].

#### 8.2.2. Bariatric Surgery

Since obesity has become a disease that leads to many other obesity-related disorders, surgical intervention is recommended for those who have clinical obesity and refractory organ failure. While historical guidelines as specified by the National Institutes of Health (NIH) rely strictly on a BMI ≥ 40 kg/m^2^ (or ≥35 kg/m^2^ with comorbidities) to qualify patients, the modern paradigm prioritizes the severity and reversibility of adipose-driven organ damage [281]. These surgical interventions involve a reduction in the size of the gastric pouch, which affects eating behaviors through endocrine and neural signalling, appetite reduction, and increased satiety. These effects, in addition to an increase in physical activity, increase and maintain the weight loss [252]. The following are the most popular surgical interventions for weight loss.

##### Sleeve Gastrectomy (SG)

This procedure is a popular bariatric surgery that consists of the removal of approximately 75–80% of the stomach, resulting in a tubular gastric sleeve. This surgery is effective for significant weight loss and improvement in obesity-related comorbidities. SG results in the loss of approximately 50% to 60% of the weight 2 years after surgery. Studies have shown that SG improves type 2 diabetes, dyslipidemia, and hypertension [253]. However, this type of surgery may lead to some complications, such as nutritional deficiencies and gastroesophageal reflux disease (GERD), due to the small size of the stomach [282].

##### Roux-en-Y Gastric Bypass (RYGB)

Characterized by the creation of a small pouch and rerouting the small intestine into this pouch. Patients are expected to lose about 70% of their excess body weight within 2 years post-surgery [255] and may maintain a substantial portion of this weight loss even 10 to 15 years post-surgery. It is known to significantly decrease body weight and to improve obesity-related diseases such as T2DM, hypertension, and dyslipidemia [254]. A previous study indicates that RYGB is more effective than SG in the long-term remission of T2DM and other metabolic disorders [254].

##### Adjustable Gastric Banding (AGB)

The procedure involves placing an adjustable silicone band around the upper part of the stomach to create a small gastric pouch, which restricts the amount of food that can be consumed at one time and promotes a feeling of fullness. Studies have shown that AGB can lead to significant weight loss and improvements in obesity-related comorbidities; however, the long-term success of AGB can be variable, with some patients experiencing complications such as band slippage, erosion, or the need for reoperation [257]. Despite these challenges, AGB remains a viable option for weight loss in selected patients, particularly those who prefer a less invasive procedure compared to other bariatric surgeries [258].

##### Elipse Gastric Balloon(s)

Intragastric balloons (IGBs) are a minimally invasive and reversible therapy for weight loss. The Elipse™ balloon is swallowed in the form of a capsule and then filled with liquid once it reaches the stomach. The balloon remains in the stomach for about 16 weeks, after which it naturally deflates and is excreted from the body. By occupying space in the stomach, the balloon helps patients feel fuller faster and reduces their overall food intake [259]. Clinical studies have shown that the Elipse™ balloon can lead to significant weight loss and improvements in metabolic parameters such as blood sugar levels and cholesterol [260]. The procedure is generally well-tolerated, with common side effects including nausea and abdominal discomfort, which typically resolve within a few days [261].

## 9. Strategies for Losing Weight in Children

Preventive strategies, particularly in children, are critical to reducing obesity rates and mitigating long-term complications. Although dietary and lifestyle modifications remain the cornerstone of obesity prevention or treatment, recent advances have introduced highly effective pharmacological options to complement bariatric surgery. Prevention of childhood obesity begins in the pre-conceptional period and during pregnancy. Preventive strategies can be implemented during the breastfeeding period. The composition and properties of human milk are known protective factors, and low-protein formulas are associated with a lower risk of obesity at 6 years. During the post-weaning period, low-carbohydrate (10–30% of total caloric intake) and low-fat (18–40% of total caloric intake) diets have demonstrated short-term efficacy [283]. The Mediterranean diet is recommended as a protective dietary approach in the most recent guidelines. It promotes increased consumption of dietary fiber, antioxidants, and long-chain fatty acids. Adherence to the Mediterranean diet is associated with higher levels of circulating vitamin D, a nutrient particularly deficient in obese children and adolescents, due to the significant impact of fat accumulation on its metabolism [284].

New medications, such as incretin therapies, including glucagon-like peptide-1 receptor agonists (GLP-1RA), have demonstrated remarkable efficacy in promoting weight loss, providing new insights into obesity-related conditions. Weight loss medications have low popularity, high costs (usually not covered by the national health care systems), and concerns regarding their safety. However, recent data on weight loss medications show promising results in adolescents [285,286]. A recent meta-analysis included 11 RCTs with 1024 patients with obesity, aged from 6 to 19 years old, showed that GLP-1 receptor agonists significantly decreased BMI z-score (MD −0.33; 95% CI −0.47 to −0.20; *p* < 0.01). GI symptoms were the most frequent adverse event (RR 1.52; 95% CI 1.09 to 2.12; *p* < 0.01) [287]. Beyond the GI side effects, pediatric and adolescent utilization of long-acting GLP-1 receptor agonists requires specialized clinical monitoring. In accordance with formal 2024 regulatory safety advisories from the FDA and EMA, long-term administration (>1 year) in developing cohorts carries a potential risk of delayed bone mineral density accrual and growth velocity deceleration, necessitating routine height and dual-energy X-ray absorptiometry (DEXA) monitoring [288,289].

Before implementing physiological escalations, family-based behavioral interventions (FBBIs) represent the foundation of pediatric care. In youth populations, dietary trajectories are profoundly modulated by a complex matrix of family dynamics, household food architecture, school micro-environments, and peer relationships. The family unit serves as the primary clinical environment, bearing the core responsibility to optimize home food availability, introduce stimulus control, and establish gradual, sustainable behavior modifications. Structured dietary counseling serves as a critical clinical instrument within this framework, enabling the precise integration of comprehensive dietary assessments alongside patient education and nutritional literacy. Ultimately, embedding the family structure and the broader environmental landscape into pediatric obesity management algorithms is an absolute clinical imperative to ensure therapeutic durability [290].

The use of bariatric surgery has shown promising results in severely obese adolescents, with evidence of long-term efficacy and good safety. Laparoscopic sleeve gastrectomy (SG) and Roux-en-Y gastric bypass (RYGB) are the most frequently performed procedures in adolescents. The maintenance of weight achieved after surgery is satisfactory from 3 years up to 12 years post-surgery. Moreover, bariatric surgery’s metabolic impact is widely acknowledged, with rates of T2D remission reaching up to 86% in adolescents compared to 53% in adults [291].

The management of pediatric obesity has undergone a paradigm shift from a conservative “watchful waiting” approach to early, intensive, multimodal treatment. The landmark 2023 American Academy of Paediatrics (AAP) Clinical Practice Guideline recommends prompt initiation of intensive health behavior and lifestyle treatment (IHBLT), defined as at least 26 h of face-to-face, family-based, multicomponent intervention over 3–12 months, as the cornerstone of therapy. Importantly, the guideline advocates escalation to pharmacotherapy in adolescents (≥12 years) with obesity when lifestyle intervention alone is insufficient and recommends referral for metabolic and bariatric surgery in adolescents aged ≥13 years with severe obesity, reflecting recognition of obesity as a chronic, progressive disease rather than a lifestyle disorder [292].

More recently, as in adults, the 2025 Lancet Diabetes & Endocrinology Commission, although it is not a treatment guideline, emphasizes that management should be individualized according to clinical consequences rather than BMI alone, promoting comprehensive assessment of metabolic, cardiovascular, orthopedic, respiratory, and psychosocial complications before therapeutic escalation. This framework supports a precision medicine approach while maintaining lifestyle intervention as first-line therapy [55].

Concurrently, randomized clinical trials have substantially expanded the evidence supporting pharmacological treatment in adolescents. In the STEP TEENS trial, once-weekly semaglutide 2.4 mg combined with lifestyle intervention produced a 16.1% reduction in BMI after 68 weeks compared with a 0.6% increase in the placebo group, with nearly three-quarters of participants achieving at least 5% weight reduction. Similarly, daily liraglutide 3.0 mg demonstrated a significantly greater reduction in BMI standard deviation score than placebo after 56 weeks in adolescents [293]. More recently, liraglutide also showed efficacy in children aged 6 to <12 years, producing significantly greater BMI reduction than placebo when combined with lifestyle intervention [294], suggesting that pharmacotherapy may become available for younger pediatric populations pending further long-term safety data [295]. Overall, these findings support the integration of GLP-1 receptor agonists into multidisciplinary obesity management for carefully selected pediatric patients while reinforcing that lifestyle modification remains the foundation of treatment.

## 10. Discussion

Obesity in the 21st century is now widely acknowledged as a chronic and multifactorial disease, extending beyond the traditional focus on BMI. The clinical understanding of obesity has evolved to include functional impairments, associated comorbidities, and broader social and environmental influences. Factors such as genetics, lifestyle, socioeconomic status, and urban infrastructure, collectively referred to as the exposome, are now seen as significant contributors to obesity risk [296].

Despite the continued use of BMI as a convenient epidemiological tool, its limitations have become increasingly apparent. BMI fails to distinguish between lean mass and fat mass, often misclassifying individuals with high muscle content or underestimating fat accumulation in those with lower muscle mass [297]. These inaccuracies are particularly relevant in pediatric, elderly, and athletic populations. As a result, the clinical utility of BMI in personalized obesity management is limited, necessitating more accurate methods for assessing body composition [298].

Alternative anthropometric measures, such as waist circumference (WC), waist-to-hip ratio (WHR), and waist-to-height ratio (WHtR), provide better estimates of visceral fat and cardiometabolic risk [299]. Imaging techniques like computed tomography (CT) and magnetic resonance imaging (MRI) have also gained traction in research settings to assess visceral adiposity, although standard clinical cut-offs remain underdeveloped [300].

The recognition of distinct obesity phenotypes, such as MHO, has further refined our understanding. Individuals with MHO may lack typical metabolic complications but still face long-term risks, including inflammation, joint problems, and reduced physical function. This suggests that even in the absence of overt metabolic dysfunction, obesity can negatively impact quality of life and health outcomes [52].

Modern definitions also frame obesity as a disease rather than a moral failing. For example, The Lancet Diabetes & Endocrinology Commission has introduced a transformative framework for understanding obesity not as a personal failing, but as a chronic, systemic disease. This shift in terminology and diagnostic criteria carries profound implications for clinical care, public health policy, and societal attitudes. Traditional terminology relies mostly on the BMI, which may misclassify metabolically unhealthy subjects with normal BMI or ignore fat distribution and organ dysfunction. However, the new framework introduces objective criteria such as adipose tissue dysfunction, organ impairment, and waist-to-hip ratio to diagnose “clinical obesity”, which explains the need to introduce new terminology to differentiate between clinical and pre-clinical obesity [151].

Importantly, obesity intersects with broader structural and social determinants of health. Access to healthy food, opportunities for physical activity, and social stigma all shape the lived experience of obesity and influence outcomes [301]. A patient-centered, multidimensional approach that integrates both individual care and policy-level interventions is essential.

Current research also addresses emerging topics such as adipose tissue dysfunction, which contributes to systemic inflammation and metabolic disturbances, and sarcopenic obesity, where fat accumulation coexists with muscle loss, increasing frailty and metabolic risk [302]. Additionally, childhood obesity, the obesity paradox, and intersectionality (how race, gender, and socioeconomic status shape obesity risk and treatment response) represent areas of growing complexity in both definition and management [303].

The integration of pharmacological therapies such as GLP-1 receptor agonists and metabolic surgery has revolutionized obesity management by enabling substantial and sustained weight loss. Yet, the persistent challenge of long-term weight maintenance underscores the need for a personalized, multimodal approach. This strategy combines behavioral modification, tailored nutrition plans, physical activity, pharmacotherapy, and, when appropriate, surgical interventions. Each modality addresses distinct physiological and psychological drivers of obesity, and their synergy enhances adherence, mitigates weight regain, and improves metabolic outcomes. Importantly, individualized care plans that evolve over time and include ongoing support are critical to sustaining weight loss and reducing obesity-related comorbidities.

Beyond established strategies such as dietary modification, physical activity, behavioral interventions, and pharmacotherapy, natural compounds and plant-derived bioactive substances have emerged as promising adjunctive tools for obesity management. Preclinical studies demonstrate that major phytochemical classes, including polyphenols, alkaloids, saponins, and terpenes, target key obesity-related pathways by inhibiting digestive enzymes (e.g., pancreatic lipase), suppressing adipogenesis via downregulation of PPARγ and C/EBP transcription factors, enhancing insulin signaling through PI3K/AKT and GLUT4 translocation, and activating energy-sensing pathways such as AMPK and SIRT1, alongside reductions in oxidative stress and inflammation. These mechanistic actions provide a strong biological rationale for translation into human interventions. Consistently, recent randomized clinical trials report improvements in body weight, BMI, visceral adiposity, glucose homeostasis, lipid profiles, hepatic markers, and adipokine regulation following supplementation with compounds such as curcumin, green coffee bean extract, licorice, fiber blends, berberine, Camu Camu, and other functional food-derived extracts. Importantly, several clinical outcomes—such as improved insulin sensitivity, reduced hepatic steatosis, modulation of adipokines, and favorable lipid changes closely mirror pathways identified in preclinical models, supporting a mechanistic continuum from cellular and animal studies to human physiology. While variability in efficacy across compounds and populations highlights the need for standardized formulations, longer interventions, and precision nutrition approaches, the convergence of mechanistic and clinical evidence suggests that nutraceuticals may complement lifestyle and pharmacological therapies, offering generally safe, multi-target strategies to address obesity and its associated metabolic complications.

Contemporary evidence supports the concept that obesity is characterized by a dysregulated biological “set point” for body weight, mediated by complex interactions between hypothalamic appetite-regulating pathways, adipose-derived hormones, gut peptides, and peripheral metabolic signals. Weight loss triggers compensatory neuroendocrine adaptations, including reductions in circulating leptin and insulin, increased orexigenic signaling, suppression of energy expenditure through adaptive thermogenesis, and enhanced food reward, all of which promote weight regain. These physiological responses explain why obesity behaves as a chronic, relapsing disease and provide a mechanistic rationale for sustained lifestyle support and, when indicated, long-term anti-obesity pharmacotherapy or metabolic surgery rather than short-term weight-loss interventions alone [304].

## 11. Conclusions

In conclusion, the clinical paradigm of obesity has undergone a transformative shift, redefining it from simple anthropometric measures to a chronic, progressive, and multisystem disease. Moving beyond the historical BMI-centric model, the implementation of the 2025 Lancet Commission framework establishes a crucial distinction between preclinical and clinical obesity. This phenotypic approach allows for precise risk stratification and multi-tiered care. Where the preclinical tier prioritizes lifestyle modifications and bioactive nutraceuticals to halt metabolic degradation before organ dysfunction occurs. Conversely, clinical obesity demands intensive escalations and potent anti-obesity medications or metabolic surgery to alleviate comorbidities.

Crucially, emerging evidence shows that weight reduction is linked to strong neuroendocrine compensatory adaptations driving a rapid biological rebound upon treatment cessation. This confirms that managing obesity requires continuous, long-term therapeutic maintenance rather than short-term interventions. Furthermore, addressing the rising crisis of pediatric obesity requires early, family-based behavioral interventions complemented by regulatory food environment policies and carefully monitored pharmacotherapies to prevent early vascular damage. Ultimately, achieving sustained metabolic health and overcoming global health disparities requires a synchronized model of care. This model must combine individualized precision medicine models with systemic pricing reforms and structural policies that target the broader commercial and socioeconomic determinants of this global epidemic.

## Figures and Tables

**Table 1:** Different types and main characteristics of obesity.

Obesity Types	Main Characteristics	References
Metabolically healthy obese (MHO)	Obesity lacks metabolic diseases. Less visceral adipose tissue, smaller adipocytes, and lower chronic metabolic inflammatory profile.	[44,45,46,47,48]
Metabolically abnormal obese (MAO)	Subjects are overweight or obese with metabolic diseases. Significant difference between MAO and MHO in levels of postprandial blood glucose, high-density lipoprotein cholesterol, insulin, and triglycerides.	[49]
Sarcopenic obesity	Characterized by a reduction of lean mass. Associated with predicting factors: age, low physical activity, smoking, low socio-economic status, pulmonary disease, and atherosclerosis. Accumulation of body fat and a decrease in skeletal muscle mass and muscle strength.	[50,51]

**Table 2:** Behavioral, dietary, and lifestyle interventions for preclinical obesity.

Intervention	Description and Core Mechanisms	Distinct Advantages	Limitations	References
Physical activity	Body movement by skeletal muscles requires energy expenditure; recommended: 150 min moderate activity or 75 min vigorous activity for adults	Weight loss; preservation of lean mass during weight loss and subsequent maintenance of weight loss	Fatigue and burnout; heart risk with high-intensity activities; financial cost; and time commitment	[20,152]
Diets				
Ketogenic diet	Severe restriction of carbohydrates (≤50 g/day or <10% of total daily energy) 70–75% fat, 20% protein	Significant drop in visceral fat mass, rapid improvement in HbA1c, fasting insulin, and triglycerides	Headaches, fatigue, nausea, dizziness, kidney stress, and constipation	[153,154,155]
Very low-calorie diet	Total energy intake restricted to 400 to 800 kcal/day, achieved through complete formula meal replacements (soups, shakes, and bars)	Rapid initial weight loss, long-term weight maintenance, and loss of fat mass	Loss of lean mass and risk of tissue stress; contraindicated in youth (%95 experience acute adverse events as negative nitrogen balance)	[156,157,158]
High-protein diet	Protein intake > 0.8 g/kg/day (or 1.2–2.0 g/kg/day during active caloric deficits); 10–35% of total daily calories.	Maintains lean mass, enhances muscle protein synthesis, increases postprandial satiety, enhances gut microbial alpha diversity	Pressure on the kidneys; constipation; nausea; dehydration	[159,160,161]
Low-fat diet	Restriction of dietary lipids to <30% of total calories (moderate fat) or <10% (very low fat); saturated fat restricted to <7%	Lower cholesterol; lower risk of heart disease; maintenance of weight loss	Hormonal imbalance; risk of compensatory overconsumption of simple refined carbohydrates	[162]
Mediterranean diet	High density of vegetables, fruits, whole grains, and fish; extra virgin olive oil as the primary monounsaturated fat source	Low CVD; low mortality; low cholesterol; low risk of heart attacks and strokes; manages blood sugar levels; lower risk of type 2 diabetes; enhances the microbiota diversity	Slower initial weight loss and requires substantial baseline cooking literacy	[163,164,165,166]
Fasting				
Intermittent fasting	Alternation between eating times and fasting times	Improves insulin sensitivity, type 2 diabetes, and blood pressure Lower cholesterol levels; reduces inflammation and oxidative stress	Hunger and cravings; fatigue and low energy; constipation, bloating, and nausea	[167,168,169,170]
Alternate-day fasting	Alternating between days of no caloric intake and days of unrestricted eating; modified versions: small caloric intake (≤600 kcal) on fasting days	Reduces body weight, BMI, total cholesterol, LDL, triglycerides, and blood pressure	Hunger and cravings; Fatigue and low energy; constipation, bloating, and nausea	[171,172]
Time-restricted eating	Specific number of eating hours (from 4 to 12), with the remaining hours spent fasting	Fat loss, improved cardiovascular health markers	Constipation and bloating; fatigue and low energy	[173,174,175]
16:8 fasting	Fasting for 16 h and eating during an 8 h window	Improves Metabolic Health; enhances insulin sensitivity; reduces blood pressure; reduces the risk of CVD.	Hunger and cravings; fatigue and low energy; digestive issues (constipation, bloating, nausea)	[176]
Ramadan fasting	Fasting from dawn until sunset	Reduces visceral and subcutaneous fat; improves GI motility; reduces GI symptoms and improves the gut microbiota	Sensation of fullness and discomfort after breaking the fast; may slow down the transit time and affect bowel movements	[177,178]

Abbreviations: CVD, Cardiovascular Disease; GI, Gastrointestinal; HbA1c, Glycated Hemoglobin; LDL, Low-Density Lipoprotein.

**Table 3:** Preclinical studies and mechanistic insight into the anti-obesity potential of natural compounds.

Compound	Source	Model	Effect	References
Polyphenols	Coffee silverskin and coffee husk	In vitro: Mouse 3T3-L1 preadipocytes and RAW264.7 Mφ cell lines	Stimulates the translocation of GLUT4, and modulates the insulin/PI3K/AKT, NF-κB/MAPK and AMPK pathways	[211]
	Grape seeds	In vivo: Rats	Activates AMPK.	[212]
	Green coffee beans	In vivo: Rats	Reduces mRNA expression of adipogenesis genes C/EBPα, SREBPs, and PPARγ. Increases SIRT1 activity in liver and leptin receptors in muscle	[213]
	Pomegranate flower	In vitro: 3T3-L1 preadipocyte cells	Inhibits PI3K/AKT activity. Downregulates PPARγ and C/EBPα Inhibits adipogenic differentiation in human adipocytes	[214]
Alkaloids	*Diospyros kaki* Fruit	In vivo: Mice	Reduces body weight Reduces fat accumulation Inhibits pancreatic lipase	[215]
	*Alstonia boonei*	In vitro: 3T3-L1 preadipocyte cells	Inhibits pancreatic lipase Antiadipogenic effect	[216]
	Garlic	In vitro: 3T3–L1preadipocytes cells	Decrease lipid accumulation Inhibits adipocytes differentiation Reduces the expression of FABP4, PPARγ, C/EBPβ, and adipsin	[217]
Saponins	Adzuki bean saponins	In vitro: 3T3-L1 preadipocyte cell line and HepG2 cell line	Improves fat metabolism Improves oxidative stress Restores mitochondrial pathway via β-catenin signaling, the PI3K/Akt/GSK3β/β-catenin signaling pathway	[218]
	Leaves of *Boussingaultia gracilis*	In vivo: Mice	Reduces liver steatosis Modulates lipid metabolism Enhances adipocytes thermogenesis Restores insulin sensitivity	[219]
Terpenes	*Callistemon citrinus*	In vivo: Rats	Decreases oxidative stress Reduces body weight Reduces fat deposition, triacylglycerol and serum glucose	[220]
	European taxon, *Ilex aquifolium*	In vivo: Rats	Reduces lipid accumulation in the liver. Antioxidant and anti-inflammatory effects	[221]

Abbreviations: AMPK, Adenosine Monophosphate-Activated Protein Kinase.

**Table 4:** Clinical studies of the potential role of natural compounds and functional foods on obesity and obesity-related conditions involving overweight and obese subjects.

Year	Study Type	No. of Subjects	Product/Intervention	Main Findings	Reference
2023	Placebo-controlled RCT	105	Green coffee bean extract (500 mg twice daily, 12 weeks)	↓ body weight (~6%), BMI (~5.6%), waist circumference, leptin, fasting glucose, HbA1c, and TSH; good safety profile	[225]
2023	Double-blind, placebo-controlled crossover RCT	30	Sesame meal extract (linoleic & oleic acids) with a high-fat meal	↓ postprandial triglycerides (−16.8% iAUC), ↓ remnant lipoproteins and LDL particles, ↑ HDL; supports pancreatic lipase inhibition	[226]
2023	Double-blind, placebo-controlled RCT	100	Sunflower seed extract (500 mg/day, 12 weeks)	↓ body fat mass, body weight, BMI, and hip circumference	[227]
2024	Double-blind, placebo-controlled RCT	272	Curcumin extract (1500 mg/day, 12 months)	↓ fasting glucose, HbA1c, BMI, HOMA-IR, leptin, ↑ β-cell function (↑ HOMA-β), ↑ adiponectin	[228]
2024	Double-blind, placebo-controlled RCT	66	Licorice extract (1.5 g/day) + low-calorie diet (8 weeks)	↓ body weight, BMI, body fat, FBS, insulin, HOMA-IR	[229]
2024	Double-blind, placebo-controlled crossover RCT	30	Camu-camu polyphenol-rich extract (1.5 g/day, 12 weeks)	↓ liver fat (−7.4%), ALT/AST	[230]
2024	Double-blind, placebo-controlled RCT	81	White kidney bean extract (Phase 2^®^, 700–1000 mg three times daily, 12 weeks)	↓ body weight, fat mass, BMI, waist, hip, and thigh circumference	[231]
2024	Double-blind, placebo-controlled RCT	112	Fiber blend (glucomannan, inulin, psyllium; 180 days)	↓ body weight, BMI, fat mass, visceral fat	[232]
2024	Double-blind, placebo-controlled pilot RCT	68	*Nitraria retusa* extract (12 weeks)	↓ body weight, BMI, body fat, and TG	[233]
2024	Comparative RCT	50	*Gymnema sylvestre* vs. Berberine (3 months)	Berberine: greater weight, BP reduction, improved adipokine gene expression; Gymnema: better fasting glucose and insulin-resistance-related adipokines; mild GI effects	[234]
2024	Double-blind, placebo-controlled RCT	70	Berberine (1500 mg/day, 12 weeks)	↓ ALT, cholesterol	[235]
2024	RCT (4-arm parallel design)	43	Spinach thylakoid extract (5 g/day) ± high-intensity functional training (HIFT)	Improved adipokines (↑ CTRP-12, KLF-15; ↓ furin), ↓ LDL, TC, TG, ↑ HDL	[236]
2025	Double-blind, placebo-controlled RCT	108	*Hibiscus sabdariffa* extract (1000 mg/day, 12 weeks)	↓ LDL	[237]
2025	Double-blind, placebo-controlled RCT	66 (63 analyzed)	Oligomerized polyphenol from *Litchi chinensis* fruit extract (200 mg/day, 12 weeks)	↓ visceral fat area (CT-measured)	[238]
2025	Double-blind, placebo-controlled RCT	109 completers (166 enrolled)	Chromium + *Phyllanthus emblica* + shilajit or *P. emblica* alone (12 weeks) with diet & exercise	Modest improvements in vascular function, insulin sensitivity, lipid profile, and body composition; strongest effects with higher *P. emblica* and Chromium doses	[239]
2025	Double-blind, placebo-controlled RCT	96	*Cyperus rotundus* extract + piperine (500 mg + 5 mg twice daily, 90 days)	↓ body weight, BMI, waist, and hip circumference	[240]
2025	Multicenter, triple-blind, randomized 2 × 2 factorial RCT	120 per group (four groups in the ratio 1:1:1:1 (placebo + SPN, QYY + SPN, placebo + PN, and QYY + PN)	Qingyanyin granules ± press needles (12 weeks)	↓ waist circumference	[241]

Abbreviations: ALT, alanine aminotransferase; AST, aspartate aminotransferase; BMI, body mass index; BP, blood pressure; CT, computed tomography; CTRP-12, C1q/TNF-related protein-12; FBS, fasting blood sugar; FLI, fatty liver index; GI, gastrointestinal; HbA1c, glycated hemoglobin; HDL, high-density lipoprotein cholesterol; HIFT, high-intensity functional training; HOMA-β, homeostasis model assessment of β-cell function; HOMA-IR, homeostasis model assessment of insulin resistance; iAUC, incremental area under the curve; KLF-15, Krüppel-like factor-15; LDL, low-density lipoprotein cholesterol; RCT, randomized controlled trial; TC, total cholesterol; TG, triglyceride; TSH, thyroid-stimulating hormone, ↓: significant decrease, ↑: significant increase.

**Table 5:** Pharmacological and surgical interventions for clinical obesity.

Intervention	Description and Core Mechanisms	Distinct Advantages	Limitations	References
Medications				
GLP-1-based drugs	Mimic the GLP-1 hormone; increase insulin secretion; decrease glucagon release; slow gastric emptying; reduce appetite; administered through subcutaneous injections	Promote both glucose control and weight loss; some agents also reduce CVD.	Nausea; vomiting; headache; constipation or diarrhea; and injection site reactions	[242,243,244,245,246,247]
CNS stimulant/appetite suppressor	Stimulates the CNS to suppress appetite or increase satiety; an oral treatment preferably combined with behavioral therapy	Promotes rapid short-term weight loss	Insomnia; increased heart rate; elevated blood pressure; dry mouth and anxiety	[245,248,249,250]
Lipase inhibitors	Inhibits pancreatic lipase and reduces intestinal fat absorption by ~30%; it is a daily oral treatment	Works independently of appetite and encourages dietary fat modification	Oily stools and possible fat-soluble vitamin deficiencies	[251]
Surgeries				
Sleeve gastrectomy	Bariatric surgery that consists of the removal of approximately 75–80% of the stomach, resulting in a tubular gastric sleeve	Significant weight loss (50% to 60% of the weight within 2 years after surgery); improves type 2 diabetes, dyslipidemia, and hypertension	Nutritional deficiencies and gastroesophageal reflux disease (GERD) are due to the small size of the stomach	[252,253]
Roux-en-Y gastric bypass	Creation of a small pouch and rerouting the small intestine into this pouch	Significantly decreases body weight (about 70% of their excess body weight within 2 years post-surgery); maintains a substantial portion of this weight loss even 10 to 15 years post-surgery; improves type 2 diabetes, hypertension, and dyslipidemia	Deficiencies in vitamins and minerals, such as vitamin B12, iron, calcium, and vitamin D, due to reduced absorption, bowel obstruction, and gallstones	[254,255,256]
Adjustable gastric banding	Placing an adjustable silicone band around the upper part of the stomach to create a small gastric pouch	Promotes a feeling of fullness; significant weight loss and improvements in obesity-related comorbidities	Probability of band slippage, erosion, or the need for reoperation	[257,258]
Elipse gastric balloon(s)	The Elipse™ balloon is swallowed in the form of a capsule and then filled with liquid once it reaches the stomach. It remains in the stomach for about 16 weeks, after which it naturally deflates and is excreted from the body	Minimally invasive procedure; reversible therapy; faster feeling of fullness; reduces overall food intake; improves blood sugar levels and cholesterol	Nausea and abdominal discomfort	[259,260,261]

Abbreviations: CNS, Central Nervous System; CVD, Cardiovascular Disease; GERD, Gastroesophageal Reflux Disease; GLP-1, Glucagon-Like Peptide-1.

**Table 6:** Current pharmacological therapies for obesity.

Drug	Adult Dosing for Weight Management	Elimination (Half-Life)	Mean Weight Loss Efficacy	Reduction in HbA1c (%)	CVD Outcomes
Tirzepatide	Initial dose of 2.5 mg/week for 4 weeks Increase to 5 mg/week. If needed, it may be increased to 2.5 mg/week increments every 4 weeks Maximum weekly dose: 15 mg/week	5 days	Around 20.9% weight reduction at 72 weeks (SURMOUNT-1)	−2 to −2.5	Benefit: 38% reduction in composite heart failure endpoints (SUMMIT trial); non-inferior MACE profile with significant cardiometabolic risk factor reduction (SURPASS-CVOT)
Semaglutide	Initial dose: 0.25 mg/week for 4 weeks Increase to 0.5 mg/week at week 5 Increase to 1 mg/week at week 9 Increase to 1.7 mg/week at week 13 Week 17 and after, the maintenance dose is 2.4 mg/week (in case of intolerance, the dose 1.7 mg/week may be used)	6 to 7 days	Around 14.9% weight reduction at 68 weeks (STEP-1)	−1.5 to −2	Benefit: 20% relative risk reduction in MACE (SELECT trial); Neutral/Benefit for HF
Liraglutide	Initial dose: 0.6 mg/day for 1 week Increase by 0.6 mg/day at weekly intervals to reach a dose of 3 mg/day. In case of intolerance to this increase, delay dose increases for 1 extra week.	11 to 15 h	Around 8.0% weight reduction at 56 weeks (SCALE)	−0.8 to −1.5	Neutral/Benefit: Cardioprotective trends observed in standard populations (SCALE and LEADER trials)
Phentermine/topiramate	Initial dose: Phentermine 3.75 mg with topiramate 23 mg/day daily for 14 days. Increase to phentermine 7.5 mg/topiramate 46 mg/day for 12 weeks In case of weight loss < 3% of baseline weight, dose may be increased to 11.25 mg phentermine/69 mg topiramate/day for 14 days (in case of tolerance) The maximum dose is 15 mg phentermine/92 mg topiramate/day	19 to 24 h	Around 8.6% to 9.3% weight reduction in 56 weeks (CONQUER)	−1.6	Neutral: Long-term CVOT data evaluating hard MACE endpoints are currently lacking; transient heart rate increases may occur
Naltrexone/bupropion	Initial dose: 8 mg naltrexone/90 mg bupropion/day. Increase to 1 dose twice daily at week 2 Increase to 2 doses in the morning and one dose in the evening at week 3 Increase to 2 doses in the morning and 2 doses in the evening at week 4 Maximum dose: 4 tablets (32 mg naltrexone/360 mg bupropion)/day	5–21 h	Around 5.0% to 6.1% weight reduction at 56 weeks (COR-I)	−0.6 (CORE-Diabetes Trial)	Neutral: Lacks definitive long-term CVOT data due to early termination of historical safety trials
Orlistat	60 mg 3 times/day (with each main meal containing fat) Maximum dose: 180 mg/day.	1–2 h	Around 5.0% to 5.8% weight reduction at 52 weeks (XENDOS)	−0.6 to −1.7	Neutral: No direct MACE or heart failure benefits; secondary metabolic benefit derived strictly from reduced intestinal fat absorption

Abbreviations: CVD, Cardiovascular Disease; CVOT, Cardiovascular Outcomes Trial; HbA1c, Glycated Hemoglobin; HF, Heart Failure; MACE, Major Adverse Cardiovascular Events.

## Data Availability

No new data were created or analyzed in this study. Data sharing is not applicable to this article.

## Abbreviations

The following abbreviations are used in this manuscript:

ADF	Alternate-day fasting
AGB	Adjustable gastric banding
AKT	Protein kinase B
AMPK	AMP-activated protein kinase
ASCVD	Atherosclerotic cardiovascular disease
ATP	Adenosine triphosphate
BA	Bile acids
BAT	Brown adipose tissue
BeAT	Beige adipose tissue
BMI	Body mass index
CDC	Centers for Disease Control and Prevention
CI	Confidence interval
CNS	Central nervous system
CT	Computed tomography
CVD	Cardiovascular disease
DALYs	Disability-adjusted life years
EASO	European Association for the Study of Obesity
FFAs	Free fatty acids
GBD	Global Burden of Disease
GERD	Gastroesophageal reflux disease
GIP	Glucose-dependent insulinotropic polypeptide
GLP-1	Glucagon-like peptide-1
GLP-1RA	Glucagon-like peptide-1 receptor agonists
GLUT4	Glucose transporter type 4
HDL	High-density lipoprotein
HRQoL	Health-related quality of life
IF	Intermittent fasting
IGBs	Intragastric balloons
LDL	Low-density lipoprotein
MACE	Major Adverse Cardiovascular Events
MASLD	Metabolic dysfunction-associated steatotic liver disease
MASH	Metabolic-associated steatohepatitis
MAO	Metabolically abnormal obesity
MD	Mean difference
MeSH	Medical Subject Headings
MHO	Metabolically healthy obesity
MRI	Magnetic resonance imaging
MR	Mendelian randomization
NCDs	Non-communicable diseases
NEFA	Non-esterified fatty acids
NIH	National Institutes of Health
PCOS	Polycystic ovarian syndrome
PI3K	Phosphoinositide 3-kinase
PPARγ	Peroxisome proliferator-activated receptor gamma
RAAS	Renin–angiotensin–aldosterone system
RCT	Randomized controlled trial
RR	Relative risk
RYGB	Roux-en-Y gastric bypass
SELECT	Semaglutide Effects on Cardiovascular Outcomes in People with Overweight or Obesity (Clinical Trial)
STEP	Semaglutide Treatment Effect in People with Obesity (Clinical Trial Series)
SG	Sleeve gastrectomy
SIRT1	Sirtuin 1
T2DM	Type 2 diabetes mellitus
TRE	Time-restricted eating
UCP1	Uncoupling protein 1
USA	United States of America
VLDL	Very low-density lipoprotein
WAT	White adipose tissue
WC	Waist circumference
WHO	World Health Organization
WHR	Waist-to-hip ratio
WHtR	Waist-to-height ratio
